# High-Entropy Electrolytes for Lithium-Ion Batteries

**DOI:** 10.1021/acsenergylett.4c01358

**Published:** 2024-07-11

**Authors:** Qidi Wang, Jianlin Wang, Jouke R. Heringa, Xuedong Bai, Marnix Wagemaker

**Affiliations:** † Department of Radiation Science and Technology, Delft University of Technology, Mekelweg 15, 2629JB Delft, The Netherlands; ‡ State Key Laboratory for Surface Physics, Institute of Physics, Chinese Academy of Sciences, Beijing, 100190, China

## Abstract

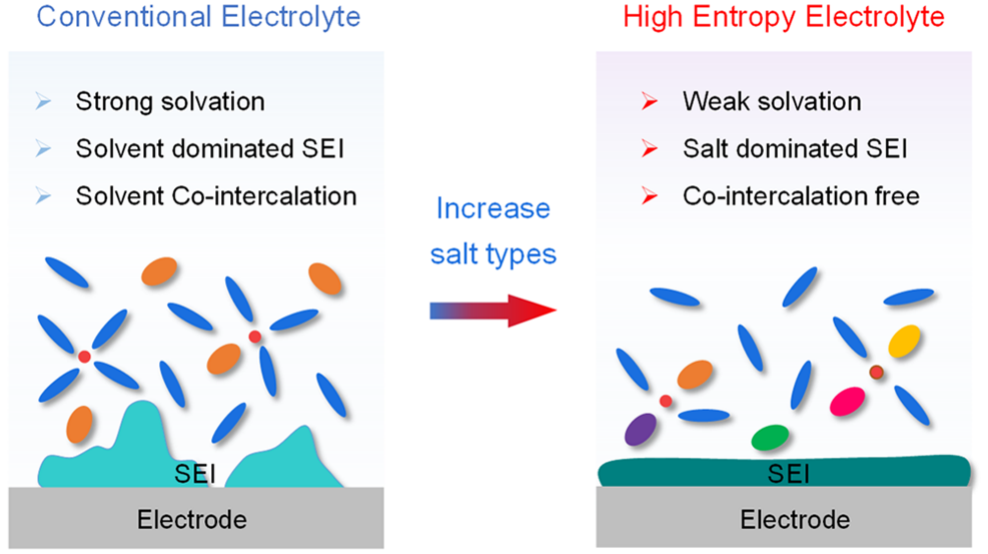

One of the primary challenges to improving lithium-ion batteries lies in comprehending and controlling the intricate interphases. However, the complexity of interface reactions and the buried nature make it difficult to establish the relationship between the interphase characteristics and electrolyte chemistry. Herein, we employ diverse characterization techniques to investigate the progression of electrode–electrolyte interphases, bringing forward opportunities to improve the interphase properties by what we refer to as high-entropy solvation disordered electrolytes. Through formulating an electrolyte with a regular 1.0 M concentration that includes multiple commercial lithium salts, the solvation interaction with lithium ions alters fundamentally. The participation of several salts can result in a weaker solvation interaction, giving rise to an anion-rich and disordered solvation sheath despite the low salt concentration. This induces a conformal, inorganic-rich interphase that effectively passivates electrodes, preventing solvent co-intercalation. Remarkably, this electrolyte significantly enhances the performance of graphite-containing anodes paired with high-capacity cathodes, offering a promising avenue for tailoring interphase chemistries.

Lithium (Li)-ion batteries (LIBs) have revolutionized society by enabling the development of portable devices, electric vehicles, and space exploration.^1^ However, the growing demand for advanced energy storage necessitates the optimization of current LIBs, with a particular focus on enhancing their energy density, safety, and cycling performance.^2−5^ In this regard, the thermodynamics and kinetics processes at the interfaces between the electrolyte and electrode are of paramount importance.^6−8^ One promising approach to address these challenges is to design advanced electrolytes that stabilize the interphases and facilitate efficient ion and charge transport within batteries.^9,10^

The most well-known example that underscores the relationship between the interphase and electrolyte is perhaps the “EC–PC disparity” in the history of LIB development.^11^ From the 1950s to the 1990s, propylene carbonate (PC) emerged as the prevailing choice for nonaqueous electrolytes, facilitating the dissolution of various Li salts.^12^ However, the development of LIB took an unforeseen turn when the introduction of the intercalation host graphite as an anode material brought the limitations of PC to the forefront. The persistent reduction decomposition of PC occurring around 0.7 V leads to detrimental consequences, ultimately contributing to the exfoliation and structural collapse of the graphite electrode.^13^ In contrast, ethylene carbonate (EC), distinguished by a mere methyl group variation in its molecular configuration, boasts a remarkable capability. It promotes the formation of a robust solid–electrolyte interphase (SEI) passivation layer, effectively curtailing electrolyte decomposition at lower potentials, thus facilitating the reversible Li^+^ (de)intercalation within the graphite framework.^14^ This divergence in performance places EC in an important role within the landscape of LIB technologies, despite its inherent drawbacks in contrast to its counterpart PC, including a comparatively elevated melting point, a restricted liquid range, and diminished anodic stability.^15^ This historical episode serves as a vivid illustration of the intricate interplay between interphase phenomena and electrolyte choices in the performance of batteries, showing the intricate trade-offs and careful considerations inherent in the quest for advanced energy storage solutions.

Over the past decades, the “Li^+^–PC solvation–co-intercalation–decomposition” model has effectively elucidated the intricate relationships among PC electrolyte compositions, the Li^+^ solvation sheath complex, and the resulting interphase chemistry on graphite anodes.^16,17^ Meanwhile, investigations into the EC–PC disparity have highlighted a critical aspect of the Li^+^ desolvation process at electrode–electrolyte interfaces. This phenomenon hinges on the competitive solvation of Li^+^ by anion and solvent molecules, ultimately determining whether an electrolyte can establish a protective interphase between EC-based and PC-based electrolytes.^11^ Consequently, using higher salt concentrations in PC electrolytes, which augments the anion population or F-donation capability due to the increased salt-to-solvent ratio, has been demonstrated as a possible way to alter the Li^+^ solvation from the PC solvent molecules to anion groups, thus reversing the observed disparity.^18,19^ However, it is important to acknowledge that resorting to concentrated electrolytes unavoidably entails trade-offs, potentially sacrificing pivotal bulk electrolyte properties like ionic conductivity, viscosity, and cost, compromising their practical applicability.^16^ In addition, researchers also investigated other strategies aimed at enhancing the interphase of graphite anodes in PC electrolytes, including the integration of film-forming additives and cosolvents (mostly ≥50% in volume),^20−25^ as well as graphite surface coatings.^26,27^ Despite efforts, these methods have fallen short of either attaining performance that rivals that of EC-based electrolytes or compromising the electrolyte properties, such as ion transport and redox stability, as well as the charge/ion transfer at interphases.^28^ Therefore, the pursuit of an approach that optimally retains the benefits of the PC solvent while avoiding the introduction of the negative effects holds great significance for both potential applications and fundamental scientific understandings.

Leveraging the vast chemical composition possibilities of electrolytes, this study presents compelling evidence that combining various commercially available salts in a propylene carbonate (PC) solution offers a straightforward yet highly efficient method to achieve the solvent-co-intercalation-free characteristic within graphite-containing anodes (Figure 1). Contrary to conventional knowledge,^18,19^ increasing the types of salts introduces the capacity to modulate the solvation interactions between Li^+^ and PC solvent toward the increased Li^+^–anion interactions (Figure 1a), achieving the same effect as the above-mentioned salt concentrated electrolytes but within a regular 1.0 M salt concentration. The intrinsically increasing diversity of solvation species by the participation of multisalt anions demonstrates a higher Li^+^ diffusion, decreased Li^+^ and PC solvent interaction, and lowered Li^+^ desolvation energy in this “so-called” high-entropy (HE)^29,30^ solvation disordered electrolyte, consisting of equimolar 0.2 M LiPF_6_/0.2 M LiTFSI/0.2 M LiFSI/0.2 M LiDFOB/0.2 M LiNO_3_ in PC. Comprehensive studies from a combination of spectroscopic techniques, including cryogenic transmission electron microscopy (cryo-TEM) and solid-state nuclear magnetic resonance (NMR) spectroscopy, indicate the electrolyte has an ability to facilitate the formation of a robust interphase, suppressing PC co-intercalation and graphite electrode degradation (Figure 1b). Consequently, this effectively resolves the incompatibility between individual salt-based PC electrolytes and graphite-based anodes (Figure 1c), resulting in significant improvements in cycling and rate performance. This study unravels the intricate solvation chemistry of the electrolytes through the incorporation of multiple salts within PC electrolytes, elucidating how this controls the characteristics of the SEI on graphite-based anodes toward high reversibility.

**Figure 1:**
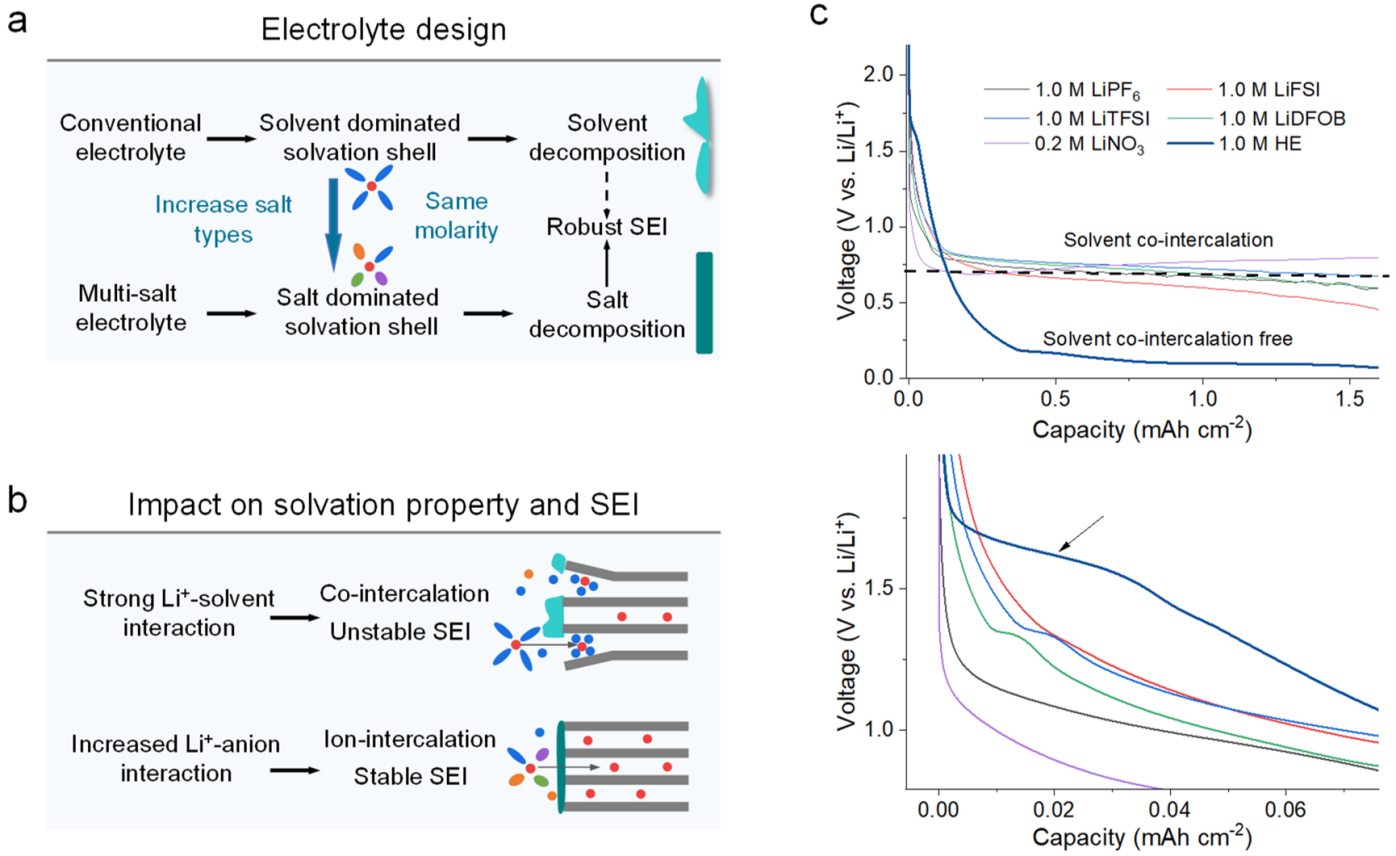
**Electrolyte design strategy and their impacts on solvation properties and interphases. a**, Electrolyte design strategy from conventional single/less-salt electrolytes to multisalt electrolytes. With an equivalent salt concentration, using diverse salts can facilitate a transition from a solvent-dominated solvation shell to a salt-dominated solvation shell. **b**, Impacts on the solvation properties and SEI on the graphite anode. The strong Li^+^–solvent interaction results in solvent co-intercalation into the graphite layers with unstable SEI. **c**, Discharge profiles of the graphite anode at 0.1C for various salt electrolytes in PC solvent (0.2 M LiNO_3_ is used because of its limited solubility). The enlarged profiles are shown at the bottom, where a short discharging plateau is observed at around 1.7 V in the HE multisalt electrolyte. The corresponding d*Q*/d*V* plots are shown in Supplementary Figure 1.

## Electrochemical Performance of Electrolytes in a Graphite Anode

**Figure 2:**
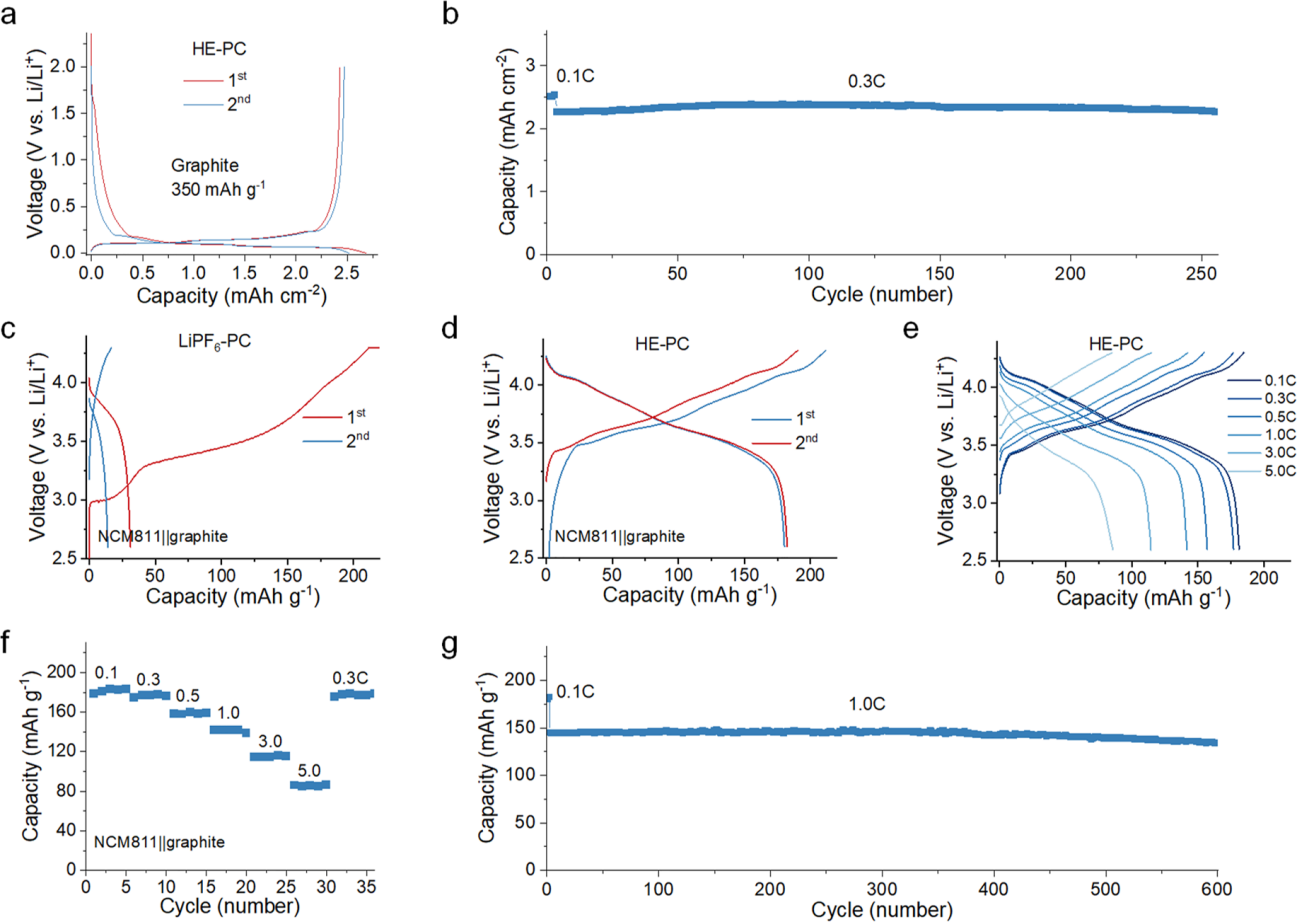
**Electrochemical performance of HE multisalt electrolyte in a graphite anode. a**, Discharge/charge profiles of a graphite anode in a graphite∥Li cell with the HE multisalt electrolyte in the voltage range 0.001–2.0 V vs Li/Li^+^. **b**, Long-term cycling performance at 0.1C for the first 3 cycles and 0.3C for the following cycles. Electrochemical performance of full cells with a graphite anode and an NCM811 cathode in **c**, LiPF_6_–PC electrolyte and **d**, HE multisalt PC electrolyte cycled between 2.6 and 4.3 V at 0.1C. **e**, The charge/discharge profiles and **f**, discharge capacity retention of full cells at various rates from 0.1C to 5.0C of HE multisalt electrolyte cycled between 2.6 and 4.3 V. **g**, Capacity retention of the NCM811∥graphite full cells cycled between 2.6 and 4.3 V. The discharge/charge rates are 0.1C for the first three cycles and 1.0C for the following cycles. The mass loading of graphite is around 2.5 mAh cm^–2^, and the N/P (anode/cathode) ratios of the full cells are in the range of 1.1–1.15.

To understand the impact of the different solvation chemistry and interphase properties on the electrochemical performance, cycling tests were conducted using graphite anodes in the HE multisalt electrolyte and a conventional LiPF_6_–PC electrolyte (used as a reference in this context). Figure 1c shows initial discharge–charge profiles of graphite∥Li cells in PC electrolytes with various single salts, where all exhibit a long plateau near 0.7 V, corresponding to the co-intercalation during the initial discharge process. The d*Q*/d*V* plots confirm the co-intercalation in conventional PC electrolytes (Supplementary Figure 1), which is held responsible for the low initial CE of around 40% as observed in the graphite∥Li cell using the LiPF_6_–PC electrolyte (Supplementary Figure 1). Interestingly, even though the solvent is identical, the cells with multiple salts strongly promote the reversibility (Figure 2a and 2b), which can be related to the salt dominated SEI formation as observed in d*Q*/d*V* plots and cyclic voltammetry (CV) measurements (Supplementary Figures 1–3 and Supplementary Note 1).

To further examine the practical feasibility, compatibility with a high-voltage cathode was evaluated (Supplementary Note 2 and Supplementary Figure 4). Full cells were assembled by combining an NCM811 (LiNi_0.8_Co_0.1_Mn_0.1_O_2_) cathode with a graphite anode. The voltage profiles of the NCM811∥graphite cell with the LiPF_6_–PC electrolyte show a first-stage slope during the first charge (Figure 2c), which is consistent with the flat plateau observed in the graphite∥Li cells, corresponding to Li^+^–PC solvent co-intercalation. This co-intercalation in the LiPF_6_–PC limits the reversible capacity to less than ∼40 mAh g^–1^ from the second cycle, which comes along with rapid capacity fading. In contrast, the full cells with the HE–PC electrolyte show a reversible capacity of about 180 mAh g^–1^ with an initial CE of approximately 84% at 0.1C (Figure 2d). The rate performance is also demonstrated by cycling at different current densities (Figure 2e and 2f), where reversible capacities of ∼181.2, 177.5, 159.7, 141.7, 116.1, and 85.6 mAh g^–1^ are obtained at rates of 0.1, 0.3, 0.5, 1.0, 3.0, and 5.0C, respectively. After the rate cycling test, a reversible capacity of around 177.2 mAh g^–1^ is delivered at 0.3C, and the battery can continue to cycle. The long-term cycling stability is further investigated (Figure 2g), resulting in a capacity retention of around ∼94.0% after 600 cycles at 1.0C, demonstrating potential application for the current LIBs.

## Solvation Chemistry of Electrolytes

**Figure 3:**
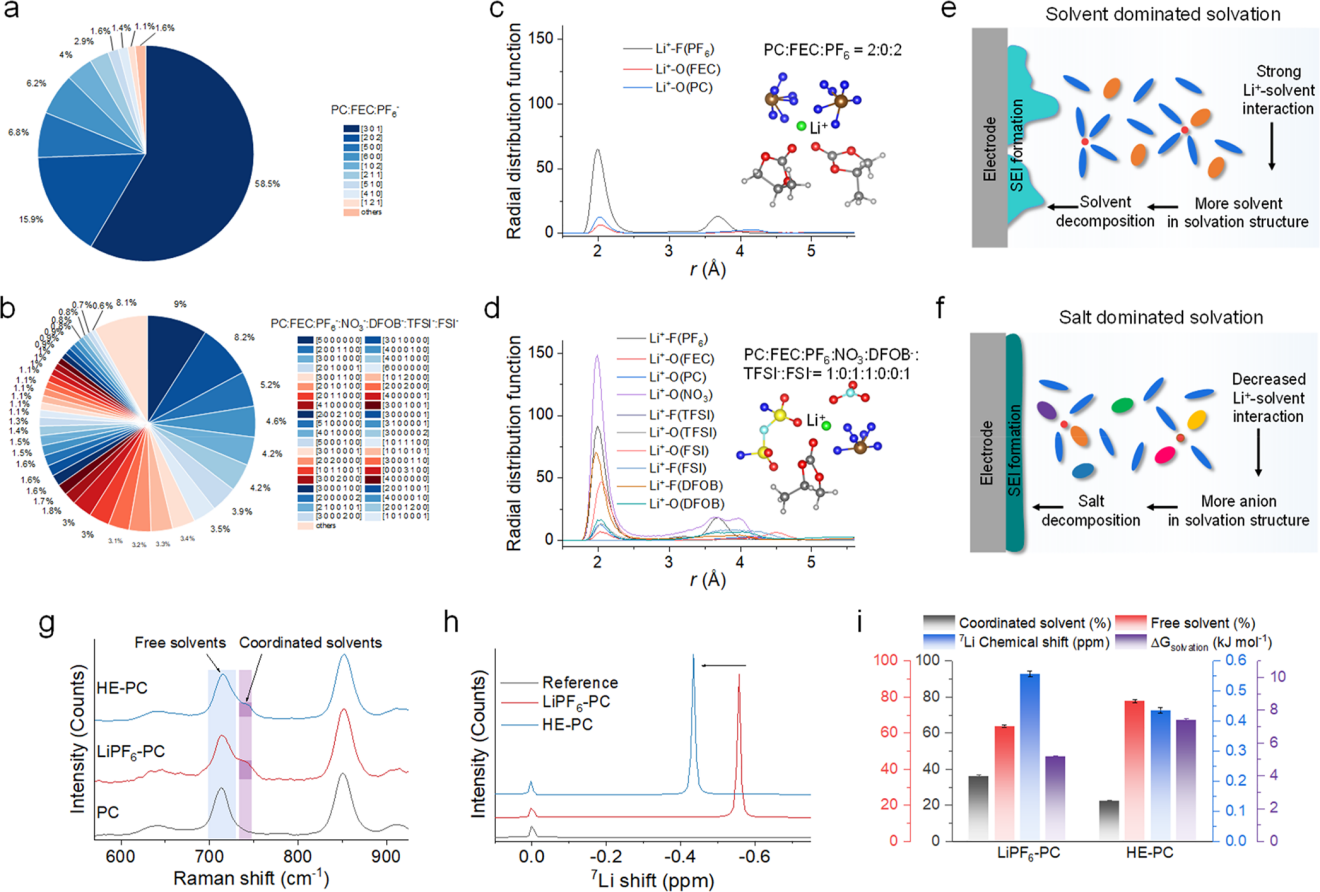
**Solvation characterizations.** Li^+^ coordination environments of **a**, single-salt LiPF_6_–PC electrolyte and **b**, HE–PC electrolyte determined from MD simulations (detailed description in Supplementary Tables 1 and 2). Simulation of the RDF for Li^+^ in **c**, LiPF_6_–PC electrolyte and in **d**, HE–PC electrolyte. **e**, Solvent-dominated solvation structure. The strong solvent-dominated solvation sheath results in an organic-rich and poorly passivated SEI, causing electrolyte consumption, low CE, and irreversible capacity loss. **f**, Salt-dominated solvation structure. This leads to an inorganic-rich robust SEI that passivates further decomposition. **g**, Raman spectra of PC solvent, single-salt LiPF_6_–PC, and HE–PC electrolyte. **h**, Liquid ^7^Li NMR spectra of LiPF_6_–PC and HE–PC electrolytes. The peaks were referenced to 1.0 M LiCl in D_2_O at 0 ppm. **i**, Comparison of the Li solvation environment properties in LiPF_6_–PC and HE–PC electrolytes. Each axis corresponds to the bar chart of the same color.

The solvation complex of electrolytes, that is, the coordination of Li^+^ to anions and PC solvent molecules, is responsible for the SEI formation and cycling reversibility.^8^ To gain more insights into the solvation structures, molecular dynamics (MD) simulations were carried out (Supplementary Figures 5–8). The various principal anion species in the HE–PC electrolyte result in a rich diversity of Li^+^ solvation environments, much more than in the single-salt LiPF_6_–PC electrolyte (Figure 3a, 3b and Supplementary Tables 1 and 2). According to the radial distribution function (RDF) results obtained from the MD simulations (Figure 3c and 3d), the solvation sheath in HE–PC electrolyte promotes the presence of more anions in the inner solvation sheath of Li^+^ compared with the LiPF_6_–PC electrolyte, leading to more salt dominated solvation configurations. The observed difference between the two electrolytes presents two typical solvation categories: solvent-dominated and salt-dominated solvation (Figure 3e and 3f). In a conventional LiPF_6_–PC electrolyte, Li^+^ is usually strongly solvated by polar solvents and most anions are excluded from the inner solvation sheath. Since the primary solvation sheath is the precursor for SEI formation, such solvation leads to solvent-derived organic-rich interphase chemistry and poorly passivated SEI, causing electrolyte consumption, low CE, and irreversible capacity loss.^16,31^ In contrast, this multisalt HE–PC electrolyte shows the salt-dominated solvation interaction, where the primary solvation sheath around the Li^+^ is dominated by anions, leading to an anion-derived inorganic-rich and robust SEI that passivates PC solvent co-intercalation and further electrolyte decomposition, enabling the good cycling of the graphite anode.^32^ This agrees with the lower amount of coordinated solvent observed in HE–PC than in LiPF_6_–PC electrolytes from Raman measurements (Figure 3g, Supplementary Figures 9 and 10).

The solvation strength is studied by ^7^Li liquid NMR spectroscopy, where the chemical shift reflects the shielding of Li^+^ as a result of the solvation environment. The HE–PC electrolyte experiences a decreased interaction between the solvation sheath and Li^+^ as reflected by the downfield chemical shift (∼0.12 ppm) as shown in Figure 3h, compared to the upfield shift for the LiPF_6_–PC electrolyte indicating more shielded Li^+^ due to the high electron density from the stronger solvation interactions.^33^ This weaker solvation observed in HE–PC electrolyte also promotes Li^+^ mobility as reflected by a higher simulated self-diffusion coefficient of 4.78 × 10^–7^ cm^2^ s^–1^ compared to LiPF_6_–PC electrolyte (1.39 × 10^–7^ cm^2^ s^–1^) (Supplementary Figure 6). In addition, the solvation energy Δ*G*_solvation_ is investigated (Figure 3i, see the method for details), which represents an overall evaluation of the binding strength between Li^+^ and solvating species (both solvent and anion). The more positive Δ*G*_solvation_ suggests a weaker solvation interaction (thus lower Li^+^–anion dissociation energy) of this HE–PC electrolyte.^34^ Altogether, these findings indicate that this HE–PC electrolyte, induced by the introduction of multiple salts in the PC solvent, can lead to a more diverse solvation environment and weaker Li^+^–PC solvent coordination that can be used to realize the solvent-co-intercalation-free property in the graphite anodes. It is worth noting that the introduction of multiple salts in a PC solvent yields results like those observed in high-salt concentration electrolytes.^16^ In both scenarios, there is a shift toward increased interaction between Li^+^ and anions, leading to the dominance of salt-induced solvation sheaths and interphases.^35^ However, they are fundamentally distinct: one involves increasing the salt-to-solvent ratio to enhance the Li^+^–anion population, while the other conceptually resembles HE alloys,^36,37^ where the presence of multiple principal elements enhances configurational diversity while maintaining the same overall salt concentration.^38−41^ This greater diversity of solvated species indicates the broadened possibility for Li^+^ ion coordination with anions, as observed in both the MD simulation and Raman measurement, because of the varying coordinating strengths and molecular structures of each salt. This result disrupts the customary local configurations between Li^+^ and the solvent; instead, it contributes to an increased potential for local solvation-disordered configurations involving salts.

## Capturing the Solvent-Co-Intercalation-Free Characteristic in Electrodes

**Figure 4:**
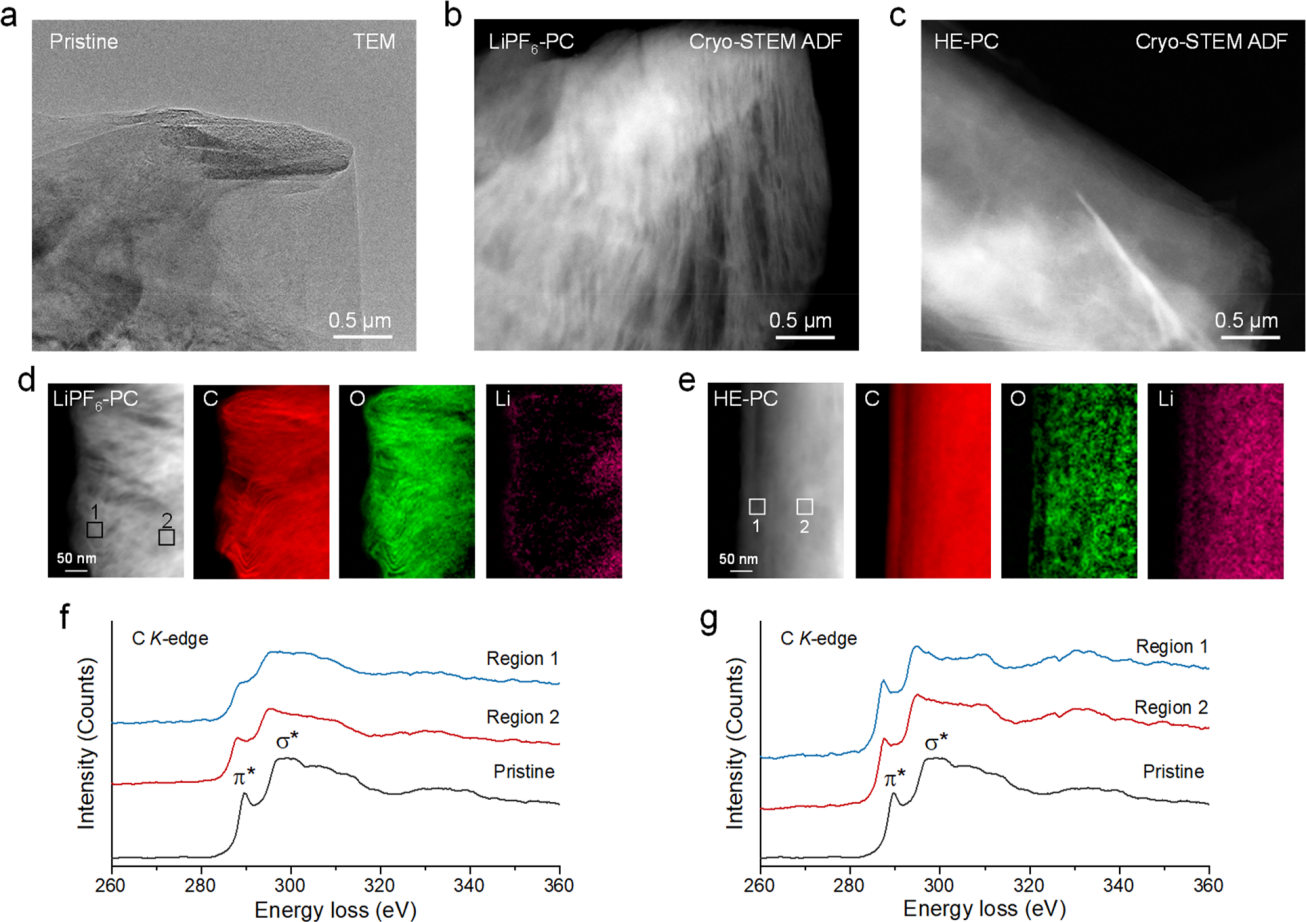
**Visualizing Li**^**+**^**–solvent co-intercalation in an electrode using cryo-STEM. a**, TEM image of pristine graphite. Cryo-STEM-ADF image of graphite cycled in **b**, single-salt LiPF_6_–PC and **c**, HE–PC electrolyte at 0.2C rate to the voltage of 0.5 V vs Li/Li^+^. Cryo-STEM EELS mappings of the graphite in **d**, single-salt LiPF_6_–PC and **e**, HE–PC electrolytes. EELS of C *K*-edge fine structure of graphite cycled in **f**, single-salt LiPF_6_–PC electrolyte and **g**, HE–PC electrolyte recorded at Region 1 and Region 2, respectively.

The TEM result of the pristine graphite material (Figure 4a) shows a smooth-edged morphology before electrochemical cycling. After discharging to 0.5 V vs Li/Li^+^ in the single-salt LiPF_6_–PC electrolyte, corresponding to the end of the Li^+^–PC solvent co-intercalation, the expanded graphite layers are observed in Figure 4b. Certain regions depict the disintegration of the graphite layers, resulting in a loss of connection with neighboring layers. In sharp contrast, good structural integrity of the electrode surface is observed in the HE–PC electrolyte (Figure 4c), without graphite exfoliation after discharging. Cryo-STEM electron energy loss spectroscopy (EELS) mappings reveal a strong oxygen signal between the carbon layers in Figure 4d, indicating the presence of the co-intercalation of the PC solvent molecules, which is more clearly observed by the stacking map of C and O (Supplementary Figures 11 and 12). By comparison, the EELS mappings show a uniform distribution of elements in graphite of the HE–PC electrolyte, and the high carbon counts are attributed to the highly reserved crystalline nature (Figure 4e).

Moreover, employing cryo-STEM-EELS analysis of the C *K*-edge can provide valuable insights into the carbon bonding environment within different regions of the graphite particle (Figure 4f and 4g). At the near surface of graphite, the EELS profile of Region 1 shows a decreased intensity of the π* peak representing the *sp*^2^ bonding, along with the broadening of the σ* peak, which indicates the transition to a more amorphous structure after cycling in the LiPF_6_–PC electrolyte (Figure 4f). At the bulk graphite, the EELS profile at Region 2 presents the pristine-like edge shapes, indicating a relatively preserved structure. Hence, parts of the graphite surface experience profound structural degradation in the LiPF_6_–PC electrolyte. As for the electrode cycled in the HE–PC electrolyte, the bonding environment of the carbon molecules within graphite (Figure 4g) shows similar π* and σ* bonding characteristics as compared to pristine graphite both in the region near the surface (Region 1) and in the region in the bulk (Region 2). The preserved graphite structure and the uniform Li and oxygen distribution suggest that Li^+^ is uniformly intercalated into the graphite layer without co-intercalation, thus highlighting the ability of the stable SEI in the HE–PC electrolyte to effectively passivate the graphite surface during the initial cycle.

## Interphase Structure and Chemistry after Cycling

**Figure 5:**
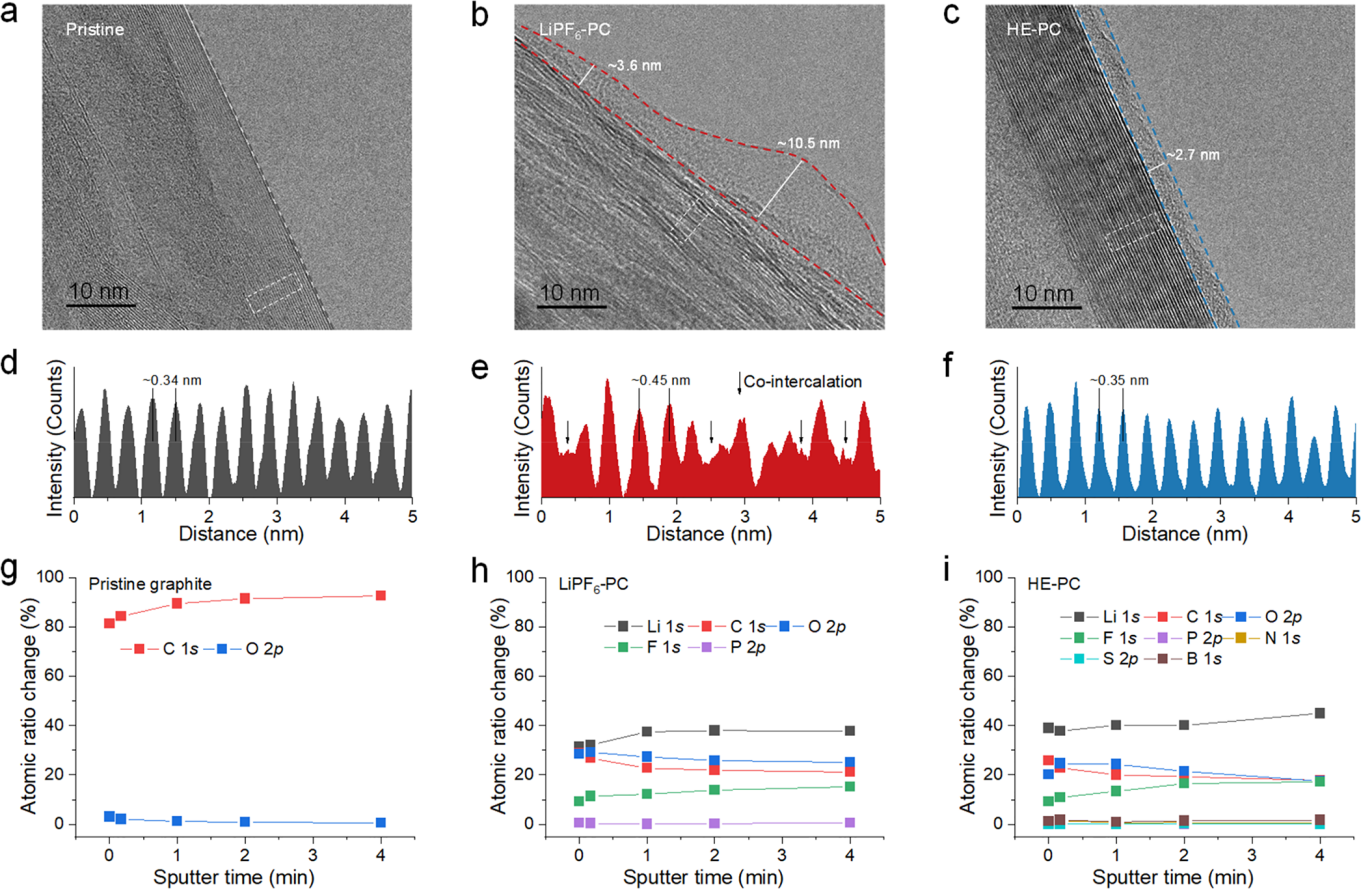
**SEI structures and chemistry. a**, High-resolution TEM images of pristine graphite. High-resolution cryo-TEM images of graphite after cycling in **b**, single-salt LiPF_6_–PC and **c**, HE–PC electrolytes. Integrated intensities of the graphite lattice in the region indicated in **a**–**c** for **d**, pristine graphite **e**, graphite cycled in the single-salt LiPF_6_–PC and **f**, and graphite cycled in the HE–PC electrolytes. Quantified atomic composition ratios of the SEI at different sputtering times for **g**, pristine graphite, **h**, graphite cycled in the LiPF_6_–PC electrolyte, and **i**, graphite cycled in the HE–PC electrolyte (from XPS spectra).

Then cryo-TEM is used to probe the nanostructure of the SEI and its interface with graphite. The pristine graphite shows a well-defined layered crystal structure in Figure 5a. After cycling, an amorphous SEI layer can be observed on the surface of graphite in both electrolytes. The uneven SEI formed in the LiPF_6_–PC electrolyte shows an average thickness larger than that formed in the HE–PC electrolyte, where the latter is uniform and homogeneous with a thickness of around 2.7 nm (Figure 5b and 5c). Moreover, the distortion and expansion of the graphite layer are also observed in the cryo-TEM results after cycling in the LiPF_6_–PC electrolyte. In comparison with pristine graphite, graphite cycled in the LiPF_6_–PC electrolyte shows an increased and irregular lattice spacing (Figure 5d and 5e), reflecting the disorder due to co-intercalation. In contrast, the crystal structure of graphite cycled in the HE–PC electrolyte is well preserved (Figure 5c and 5f).

In addition, we further studied the SEI composition using XPS measurements on graphite electrodes in the two electrolytes. The atomic concentration after different sputtering times reveals the SEI composition as a function of depth (Figures 5g–i, Supplementary Figures 13–19). For the pristine graphite electrode, the surface contains a large amount of C and a small amount of O (Figure 5g). After cycling in the LiPF_6_–PC electrolyte, the SEI shows high C and O content, while less F content indicates solvent-dominated decomposition in SEI formation (Figure 5h). In contrast to the LiPF_6_–PC electrolyte, the atomic composition of SEI in the HE–PC electrolyte shows lower C, O content and higher F content as well as N, B, and S species that originate from salt decomposition (Figure 5i). This implies that the SEI formed in the HE–PC electrolyte has more anion-derived interfacial chemistry (Supplementary Note 3). This is further confirmed by the deconvolution of the C 1*s* and O 1*s* spectra (Supplementary Figures 13 and 14). The C 1*s* spectra of pristine graphite reveal four peaks, including C—C (from graphite), C—H, C—O, and π*—π* spectra (from graphite). After cycling, the C=O species resulting from PC solvent decomposition appears. The peak intensity of C—O and C=O in the SEI from the HE–PC electrolyte is lower than that in the LiPF_6_–PC electrolyte, confirming the more inorganic-rich SEI due to the anion-dominated solvation structure, which is held responsible for passivating and thereby stabilizing the graphite electrode during cycling. Considering that the formation of a stable SEI is also an intriguing aspect when examining Li-metal anodes, Li∥Cu cells were assembled with two electrolytes, showing higher Coulombic efficiency (CE) exceeding 99% for HE–PC electrolyte (Supplementary Note 4 and Supplementary Figures 20 and 21).

## Electrode Structure Evolution upon Cycling

**Figure 6:**
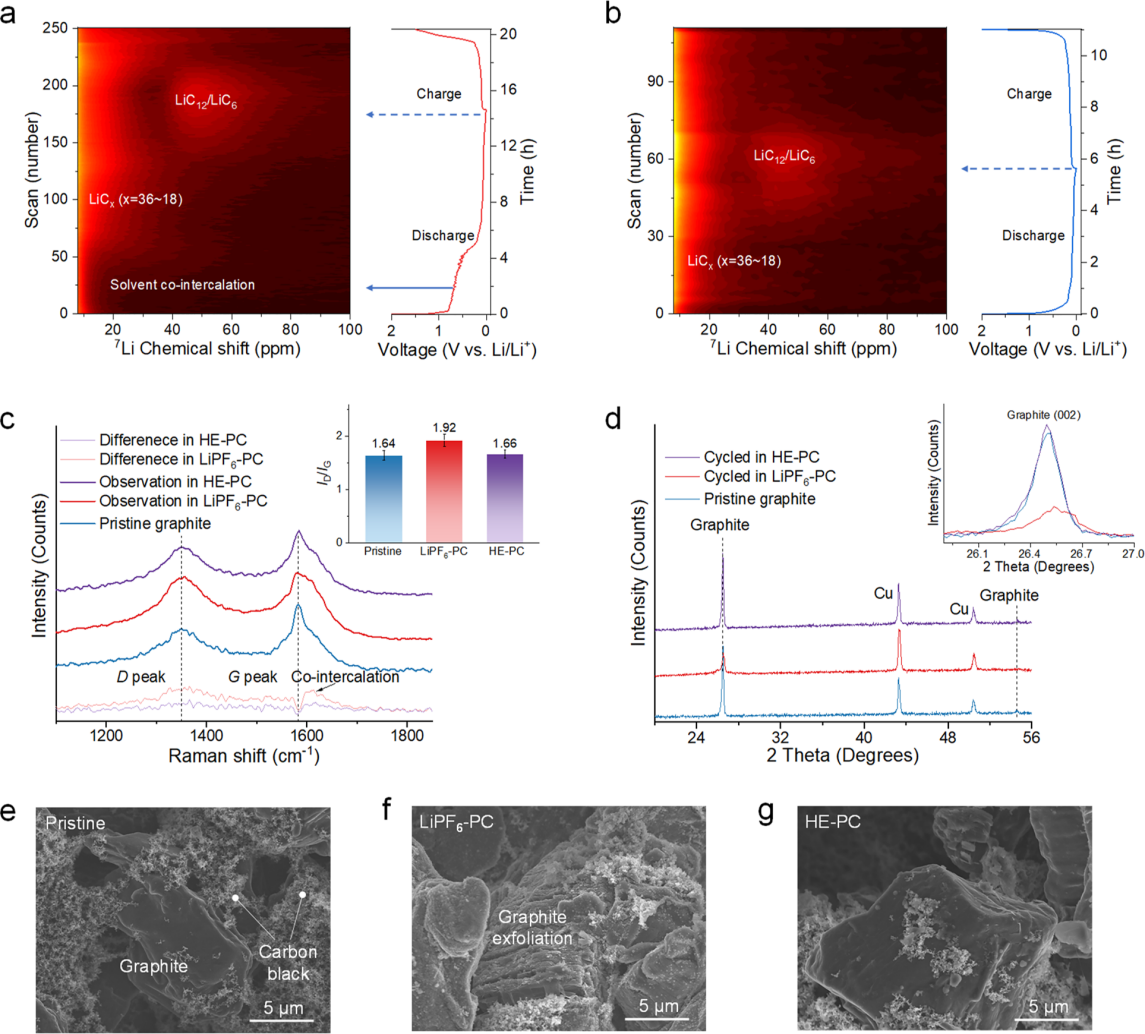
**Structure evolution of the graphite anode after cycling.** Discharge/charge profile and contour plots of operando ^7^Li NMR data of graphite∥Li cells between 0.001 and 2.0 V at 0.2C rate using **a**, single-salt LiPF_6_–PC and **b**, HE–PC electrolytes. **c**, Raman spectra of the graphite anode before (pristine graphite) and after one cycle in LiPF_6_–PC and HE–PC electrolytes. The light red and light purple lines at the bottom show the differential spectrum between the pristine graphite and cycled graphite, showing different degrees of structure degradation. The inset shows the *I*_D_/*I*_G_ ratio calculated by integrated intensity, showing different degrees of graphitization and defects. **d**, XRD patterns of the graphite anode before and after cycling in LiPF_6_–PC and HE–PC electrolytes. The inset shows a magnified image of the graphite (002) peak. **e**, SEM image of a pristine graphite anode. SEM image of the graphite anode after cycling in **f**, LiPF_6_–PC electrolyte and in **g**, HE–PC electrolyte.

The evolution of the corresponding electrode structure in various electrolytes is investigated using solid-state nuclear magnetic resonance (NMR), a potent tool offering insights into the changing chemical state and environment of specific nuclei.^42−46^ In this context, operando ^7^Li NMR is employed to observe the Li^+^–solvent co-intercalation behavior within graphite∥Li cells utilizing different electrolytes. The setup for operando NMR measurements is illustrated in Supplementary Figure 22. Figure 6a and Supplementary Figure 23 present the evolution of the ^7^Li resonance in the graphite∥Li cell utilizing the LiPF_6_–PC electrolyte, captured during the initial cycle. The extended voltage plateau attributable to solvent co-intercalation becomes evident around 0.7 V vs Li/Li^+^, during which the ^7^Li chemical shift aligns near 0 ppm. This suggests a comparable Li^+^ environment in the co-intercalated species when compared to the electrolyte, making it indistinguishable from the strong electrolyte peak also around 0 ppm. Throughout the co-intercalation process, no novel Li^+^ environment emerges, as discerned from the spectra captured at different discharge and charge stages (Supplementary Figure 24). After co-intercalation, a ^7^Li resonance emerges at around 15 ppm, growing in intensity and shifting to around 30 ppm. This observation aligns with the formation of LiC_*x*_ (18 < *x* < 36) compounds.^47^ Additionally, an extra resonance emerges at approximately 50 ppm, which is attributed to LiC_12_/LiC_6_ compounds. Concurrently, the intensity within the 30 ppm region diminishes while shifting to a lower ppm value, indicating a transformation between these species. During charging, Li deintercalation from the graphite leads to a decline in the LiC_12_/LiC_6_ resonance. Remarkably, at the end of charging at 2 V, the intensity of the resonance linked to LiC_*x*_ (18 < *x* < 36) remains significant, suggesting a substantial amount of trapped Li within the graphite. This finding elucidates the lower initial CE for the LiPF_6_–PC electrolyte. Operando ^7^Li NMR analysis of the graphite∥Li cell using the HE–PC electrolyte is also conducted for comparison, as depicted in Figure 6b and Supplementary Figure 23. As anticipated, there is no co-intercalation region observed, and the LiC_*x*_ (18 < *x* < 36) resonance emerges almost immediately upon the discharge (Supplementary Figure 25). In this instance, the resonance shift occurs more continuously compared with the LiPF_6_–PC electrolyte, suggesting a more uniform intercalation process. Furthermore, the lower intensity of the LiC_*x*_ resonance at the end of the charging process indicates superior reversibility of Li-intercalation for the HE–PC electrolyte in contrast to the LiPF_6_–PC electrolyte.

To further investigate the changes in graphite structure upon cycling, Raman spectra were conducted on electrodes before (pristine graphite) and after cycling. The results are depicted in Figure 6c and Supplementary Figure 26. The ratio of the relative intensity between the D and G bands, denoted as *I*_D_/*I*_G_, around 1350 and 1580 cm^–1^, respectively, serves as an indicator for assessing the extent of carbon structure defects. After cycling with the LiPF_6_–PC electrolyte, this ratio significantly increases to 1.92 compared to pristine graphite (1.64), signifying a more defective structure and, consequently, a reduced degree of graphitization due to the co-intercalation. In contrast, the graphite cycled with the HE–PC electrolyte maintains a consistent *I*_D_/*I*_G_ ratio of 1.66, indicating the preservation of its structure during cycling. Electrode structure analysis was further conducted using X-ray diffraction (XRD), where the patterns of the graphite electrode before and after cycling in different electrolytes were captured (Figure 6d). The (002) graphite peak at approximately 26.5° 2θ demonstrates a decrease in intensity and broadening after cycling in the LiPF_6_–PC electrolyte, consistent with interlayer spacing expansion due to Li^+^–PC co-intercalation. On the contrary, the graphite (002) peak remains unchanged after cycling with the HE–PC electrolyte, indicating structural stability. Further insights into electrode morphology and structure were obtained using scanning electron microscopy (SEM) as depicted in Figure 6e–g, with additional details in Supplementary Figure 27. The graphite anode cycled in the LiPF_6_–PC electrolyte experiences extensive exfoliation, while the graphite particles remain intact with a smooth surface after cycling with the HE–PC electrolyte. Energy-dispersive spectroscopy (EDS) (Supplementary Figures 28–30) findings reveal that the graphite surface cycled in the LiPF_6_–PC electrolyte is enriched with oxygen, pointing to a solvent-dominated SEI. Conversely, the O intensity is notably low for graphite cycled with the HE–PC electrolyte, whereas P, F, and S are more prominently present, indicating a salt-dominated SEI.

## Electrochemical Performance of Electrolytes in Silicon–Graphite Composite Anodes

**Figure 7:**
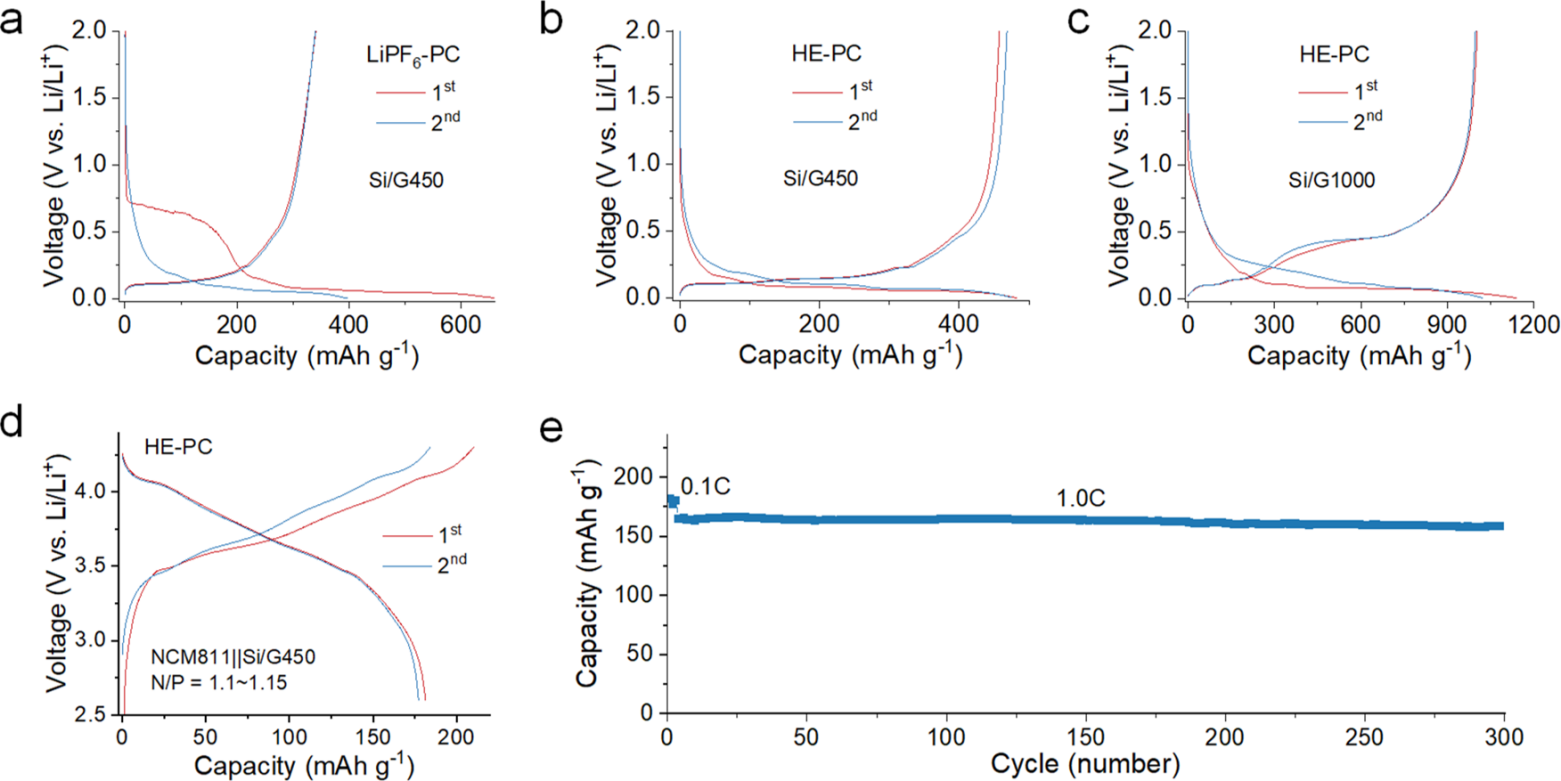
**Electrochemical performance of Si/graphite composite anodes.** Charge/discharge profiles of Si/graphite∥Li cells with a Si/G450 anode in **a**, LiPF_6_–PC electrolyte and **b**, a HE–PC electrolyte and **c**, a Si/G1000 anode in HE–PC electrolyte at 0.1C rate between 0.001 and 2.0 V vs Li/Li^+^. **d, e**, Cycling performance and corresponding voltage profiles of the full cells using the HE–PC electrolyte cycled between 2.6 and 4.3 V using a Si/G450 anode. The discharge/charge rates are 0.1C for the first three cycles and 1.0C for the following cycles. The mass loading of Si/graphite composite anodes is around 2.5 mAh cm^–2^, and the N/P (anode/cathode) ratios of the cells are in the range of 1.1–1.15.

Next, we extend the application of this HE–PC electrolyte to Si/graphite composite anodes, which offer a higher specific capacity to increase the battery energy density. Given the presence of graphite, conventional PC-based electrolytes are typically deemed incompatible. As illustrated in Figure 7a, employing the LiPF_6_–PC electrolyte in combination with a Si/graphite composite anode with a specific capacity of 450 mAh g^–1^ (Si/G450) yields a lower CE of approximately 51.0%, attributable to the co-intercalation. In contrast, utilizing the HE–PC electrolyte yields a significantly improved CE surpassing 95.0% (Figure 7b). Furthermore, an anode with a higher Si fraction and a specific capacity of 1000 mAh g^–1^ (Si/G1000) demonstrates a promising performance. It exhibits an initial CE exceeding 88.5% and maintains reversible cycling paired with HE–PC electrolyte (Figure 7c). To explore the application of the HE–PC electrolyte in cells with higher energy density, the electrochemical performance of NCM811∥Si/G450 full cells utilizing the HE–PC electrolyte is assessed (Figure 7d). The cell exhibits an initial discharge capacity of approximately 180 mAh g^–1^, accompanied by an initial CE of 86.3%. Following the initial cycles at 0.1C, the cell displays minimal degradation during subsequent cycles at a rate of 1.0C. The discharge capacity attains 162 mAh g^–1^ at the 150th cycle and 157 mAh g^–1^ at the 300th cycle, resulting in impressive capacity retentions of 97.5% and 94.5%, respectively (Figure 7e).

In summary, our study shows the effectiveness of introducing multiple salts to engineer electrolyte compositions, thereby opening avenues for the advancement of next-generation high-energy LIBs. This HE multisalt electrolyte has yielded intriguing results, particularly in realizing a reversed solvation chemistry, which enables a transformative shift from strong Li^+^–solvent solvation to enhanced Li^+^–anion interactions within the same total salt concentration. This alteration in the solvation structure bears two significant outcomes. First, it contributes to the reduction of desolvation energy, which facilitates efficient Li^+^ transport and accelerates charge transfer processes. Second, the prevalence of a salt-dominated solvation structure leads to the creation of a robust inorganic-rich SEI layer. This protective interphase acts as a barrier, effectively preventing continuous electrolyte decomposition and electrode deterioration. This strategy is realized by combining five commonly used salts in a PC solvent to formulate an electrolyte with a standard 1.0 M concentration. This approach successfully eliminates solvent co-intercalation in graphite-containing anodes, a distinct achievement not attainable in all single-salt electrolytes. Importantly, our approach departs from conventional methods. The introduction of various salts engenders solvation interactions between Li ions, solvents, and anions, diverging from common strategies such as incorporating film-forming additives or raising salt concentration to increase salt participation in solvation. The integration of multiple salts can increase the disorder (or entropy) of mixing,^36,37^ thereby expanding the realm of possibilities for Li^+^–anion complexes within the solvation sheath. Through a practical illustration involving the PC–graphite system’s inherent incompatibility, our study indicates the potential of altering solvation chemistry via mixing salts to address this long-standing challenge at electrode–electrolyte interphases. The outcomes of this approach have yielded unexpected advancements in battery performance, also as demonstrated in higher capacity Si/graphite anodes in combination with high capacity NCM811 cathodes. Further improvements could be achieved by exploring different solvent systems and incorporating novel salts. We hope our study is a catalyst not only for reevaluating the utilization of materials such as PC solvent in this context but also for charting novel avenues in advanced electrolyte chemistry and beyond.

## Supporting Information Available

Experimental procedures and computational details, supplementary note, galvanostatic charge/discharge plots, CV curves, Li^+^ self-diffusion coefficient, Raman measurement, cryo-STEM EELS, XPS spectra, operando NMR, SEM and EDS characterizations, and supplementary tables (PDF)
