# Supplementary material for: High-Entropy Electrolytes for Lithium-Ion Batteries
Source: ACS Energy Lett. 2024 Jul 11;9(8):3796–806. doi: 10.1021/acsenergylett.4c01358 (PMC11320655; doi:10.1021/acsenergylett.4c01358)
Supplement: Supplementary file 1 — nz4c01358_si_001.pdf [file nz4c01358_si_001.pdf]

Supporting information

High-Entropy Electrolytes for Lithium-Ion Batteries

Qidi Wang[1,†,*], Jianlin Wang[2,†], Jouke R. Heringa[1], Xuedong Bai[2], Marnix Wagemaker[1,*]

[1]Department of Radiation Science and Technology, Delft University of Technology, Mekelweg 15, 2629JB Delft, the Netherlands.

[2]State Key Laboratory for Surface Physics, Institute of Physics, Chinese Academy of Sciences; Beijing, 100190, China.

[†]Q.W. and J.W. contributed equally to this work.

*Corresponding author. Email: q.wang-11@tudelft.nl; m.wagemaker@tudelft.nl.

Materials and methods

Materials

Solvent of propylene carbonate (PC) was purchased from Sigma-Aldrich with battery-grade purity, which was dehydrated with a 4 Å molecular sieve (Sigma-Aldrich) to eliminate the trace water. Lithium hexafluorophosphate ($LiPF_6$), lithium bis(fluorosulfonyl)imide (LiFSI), lithium bis(trifluoromethanesulfonyl) imide (LiTFSI), lithium difluoro(oxalato)borate (LiDFOB) were obtained from Sigma-Aldrich. Lithium nitrate ($LiNO_3$, >99.9%) was purchased from Shenzhen Capchem Technology Co., Ltd and used as received. All the electrolytes were prepared by dissolving the specific amount of different Li salts in solvents in an Ar-filled glove box (H_2O < 0.1 ppm, O_2 < 0.1 ppm). Li-metal foils (thickness of 250 μm), Cu foils and Al foils were purchased from MTI Corporation. All Li-metal foils were washed 3 times with DMC solvent before use. Cu foils were immersed in diluted acetic acid for several minutes, subsequently washed by deionized water and acetone three times, separately, then they were quickly dried in the vacuum oven of glove box at room temperature.

Preparation of electrolytes

The 1.0 mol L^{-1} (M) $LiPF_6$-PC is prepared by dissolving 1.0 M $LiPF_6$ in the PC solvent. The multi-salt HE-PC electrolyte was prepared by dissolving 0.2 M $LiNO_3$, 0.2 M LiFSI, 0.2 M $LiPF_6$, 0.2 M LiDFOB, 0.2 M LiTFSI in the PC solvent. $LiNO_3$ was firstly dissolved into PC under 60-80 ^{o}C, and then the other salts were added into this mixture. All the electrolytes contain 5% FEC in volume.

Electrochemical measurements

Electrochemical cycling tests of all batteries were based on CR2032 coin cells assembled in an Ar-filled glove box (H_2O < 0.1 ppm, O_2 < 0.1 ppm) with Solupor separator manufactured by DSM Solutech, unless stated otherwise. 70 μL electrolytes were injected into each coin cell for comparison. All coin cells were tested using multi-channel battery testing systems (Land CT2001A or Lanhe G340A). For Li‖Cu cells, 14 mm diameter Li-metal foils and 16 mm Cu foils were used, with the effective area for Li-metal deposition of 1.54 cm^2. Graphite||Li cells

were tested with graphite electrode with areal capacity of 2.5 mAh cm^{-2}, and Li-metal foils were used as counter electrodes. All full cells were cycled under a 0.1C rate for three cycles before cycling at 1C rate (1C=~180 mA g^{-1}). The capacity ratio between the anode (the negative electrode) and cathode (the positive electrode), known as N/P ratio, is around 1.1~1.15. Cyclic voltammetry (CV) of Li||Cu cells with different electrolytes were conducted at a scan rate of 0.8 mV s^{-1} from -0.1 to 2.5 V vs. Li/Li^{+}.

The solvation energy was measured with the method based on ref. 1. By measuring open circuit potential (OCV) in a cell with symmetric electrodes and asymmetric electrolytes, the effects of anions, and solvents on solvation energy across varied electrolytes can be quantitatively characterized. A home-made apparatus consisting of a T-shaped flange assembled between H-cell was used for the measurement. The apparatus is divided to three chambers containing test electrolyte, salt bridge electrolyte, and reference electrolyte, respectively. Solupor separators were used as porous junctions to separate the three chambers. Two pieces of fresh lithium metal foil were used as electrodes in both sides in the H-cell. Salt bridge (3.0 M LiTFSI in DOL/DME 1:1 in volume), reference electrolyte (1.0 M LiFSI in DEC) and experiment electrolyte were put in H-cell, and each chamber was capped to prevent evaporation. Each electrode was connected to a potentiometer (Biologic VMP3) to measure the OCV, and the voltage was recorded after stabilization of a few minutes. The solvation Gibbs free energy ($\Delta G_{solvation}$) was converted from the measured H-cell OCV using equation: $\Delta G = -nFE$.

Materials characterization

Morphologies of electrodes were measured on a cold field scanning electron microscope (SEM, HITACH-S4800, SU8010) with energy-dispersive spectroscopy (EDS) for elemental analysis. Elemental composition on the surface of the electrodes was analyzed by X-ray photoelectron spectroscopy (XPS, PHI 5000 VersaProbe II) using a monochromatic Al Kα X-ray source. For depth profiling of the electrodes, argon sputtering for the XPS depth-profiling was carried out with beam energy of 1 kV and current of 0.5 μA. Peaks were fitted using MultiPak software calibrated with respect to carbon (284.8 eV). The above morphology and composition characterization were performed with cells being disassembled after specific cycles in an Ar-

filled glove box and rinsed with pure DMC solvent three times to remove residual electrolyte, followed by drying in a glove box for several hours at room temperature to remove the residual solvent. Then these electrodes were transferred into the vacuum transfer boxes for measurements to avoid air exposure. XRD was performed using a Bruker D8 Advance diffractometer equipped with a Cu Kα radiation source (λ_1 = 1.54060 Å, λ_2 = 1.54439 Å at 40 kV and 40 mA) and a LynxEye_XE detector. Raman spectroscopy was measured by Micro-laser confocal Raman spectrometer (Horiba LabRAM HR800 spectrometer) equipped with an Olympus BX microscope and an argon ion laser (532 nm) at room temperature. All the electrolytes were hermetically sealed in quartz cuvettes in a glovebox before measurement.

Conventional and cryo-TEM experiments were performed on a scanning transmission electron microscope (STEM) (JEM-ARM300F, JEOL Ltd.) operated at 300 kV with a cold field emission gun and double Cs correctors. During image acquisition, the corresponding electron dose flux (units of number of electrons per square angström per second, e^- Å^{-2} s^{-1}) was recorded. Conventional STEM images were taken with a dose rate of over 1000 e^- Å^{-2} s^{-1} with an exposure time for each image of several seconds. Cryo-TEM images were obtained with an exposure time for each image of around 0.3 s with built-in drift correction function in GMS3. Cryo-TEM images were taken with an electron dose rate of 50-500 e^- Å^{-2} s^{-1}. Short-exposure single-frame shots were used to estimate the defocus and make it as close as possible to Scherzer defocus. The EELS spectrum images were carried out with a camera length of 20 mm, and a pixel dwell time of 10 ms. Energy drift during spectrum imaging was corrected by centering the zero-loss peak to 0 eV at each pixel. Elemental maps were computed through a two-window method in a pre-edge window fitted to a power-law background and a post-edge window of 50-200 eV on the core-loss signal. Analysis of the spectra has been performed in Digital Micrograph.

For cryo-TEM preparation of graphite, graphite||Li cells were cycled at 0.2C and then disassembled in glovebox. After rinsing, a small piece of electrode was sealed in an airtight container with pure DMC inside. Then the sealed airtight container was taken out from glovebox and the sample was dispersed for three minutes by ultrasonic method. After that, the dispersed graphite was dropped on the TEM grids in glovebox and loaded into the cryo-TEM holder for further measurement. The same specialized shutter was also used to prevent air

exposure. All cryo-TEM images are taken at around -170 ^{o}C to reduce beam damage. For the conventional TEM experiments, the dispersed graphite sample was dropped on a copper grid, dried for an hour in a vacuum and loaded into the double-tilt holder. Then the TEM images were recorded at room temperature.

Liquid NMR spectra were recorded with an Agilent 400 MHz DD2 NMR spectrometer with 5 mm ONE NMR Probe at room temperature, which worked at 155.5 MHz on ^{7}Li. The chemical shift values are given in ppm. ^{7}Li chemical shift was referenced to the standard solution: 1 M LiCl in D_2O for ^{7}Li (0 ppm). All referenced solutions are measured in the enclosed internal capillary in D_2O. During measurement, all electrolytes were sealed into 5-mm Pyrex capillary tubes with PTFE caps, and then was inserted into an NMR tube containing the external standard solutions. Mestrelab Research Mnova was used for data processing. Operando solid-state NMR measurements were conducted on a wide-bore Bruker Ascend 500 system equipped with a NEO console with a magnetic field strength of 11.7 T and a ^{7}Li resonance frequency being 194.37 MHz using a solenoidal Ag-coated Cu coil. Operando static ^{7}Li NMR measurements were performed using an automatic-tuning-and-matching probe (ATM VTX operando WB NMR probe, NMR Service) at room temperature which can allow for an automatic recalibration of the NMR radio-frequency (rf) circuit during an operando electrochemistry experiment. A highly shielded wire with low-pass filters was attached to the probe for electrochemical measurement, which could minimize the interferences between NMR and the electrochemistry circuit. Single pulse with a $\pi/2$ pulse of 3 μs and recycle delay of 8.0 s was applied to acquire the 1D static spectrums. The electrochemical cell was simultaneously controlled by a Maccor battery testing system. A plastic capsule cell made from polyether ether ketone (PEEK) was used for the operando NMR experiments. The cells were assembled using graphite and Li-metal foils as working and counter electrodes with both a piece of Celgard and a piece of Solupor separator as a separator. The operando capsule cell was aligned in an Ag-coated Cu coil with graphite and lithium metal foil electrodes oriented perpendicular to B_0 and parallel with respect to the B_1 rf-field. During the static ^{7}Li NMR measurements, the cells were cycled at 0.2C in the voltage range of 0.001-3 V. The chemical shift of ^{7}Li was referenced to 1 M aqueous solution of LiCl at 0 ppm. The spectra were processed in the Bruker Topspin

software, using the automatic phase and baseline correction. Mestrenova was used for data processing and analysis.

Molecular dynamics

Classical molecular dynamics (MD) simulations were conducted on electrolyte systems with different Li salts using the Groningen Machine for Chemical Simulations (GROMACS)[2-5]. Molecular forces were calculated using the generalized Amber force field (GAFF)[6]. Topology files and bonded and Lennard–Jones parameters were generated using the acpype script[7]. The optimization of the molecular geometries was performed via gaussian 09 package at a level of B3LYP/6–311G+ (d, p). Partial charges were computed by fitting the molecular ESP at the atomic centers with the Møller–Plesset second-order perturbation method with the correlation-consistent polarized valence cc-pVTZ basis set[8]. Simulation boxes were constructed using the software Packmol[9] with the dimensions of 10×10×10 nm^3. The single salt model contains 1000 PC, 50 FEC, 90 $LiPF_6$, while the multiple salts system contains 1000 PC, 50 FEC, 18 $LiPF_6$, 18 $LiNO_3$, 18 LiDFOB, 18 LiTFSI, 18 LiFSI. A cut-off distance of 1.0 nm was chosen for the Lennard-Jones interactions. The particle mesh Ewald method was used to calculate electrostatic interactions, with a Fourier spacing of 0.16 nm. Periodic boundary conditions were applied in all directions. The energy minimization on both simulation boxes was first performed using the steepest descent method. A time-step of 2 fs was chosen for the MD simulations performed after this point. Subsequently, both systems were equilibrated at room temperature using canonical ensemble simulations. Then, isothermal-isobaric ensemble simulations at 300 K were then performed to obtain the correct volumes of both systems. The final 20 ns of the production run were used for the analysis of the radial density functions and the diffusivities of the lithium ions, which were computed using the MDAnalysis package[10]. The solvation structures for both electrolytes were analyzed with the final 1 ns using the script based on MDAnalysis package. Visualizations were generated with VESTA.

Supplementary Note 1

Salt dominated interphase formation

Since salt decomposition takes place around ~1.5 V, whereas the decomposition of cyclic carbonate solvents occurs around 0.5 V[11], the SEI formation from salt decomposition turned out to be one of the factors to support the improved cycling stability, as indicated by a clear peak around ~1.7 V during the initial stages of the first discharge in the *d*Q/*d*V plots (Supplementary Fig. 1). This is also supported by the redox peaks in cyclic voltammetry (CV) measurements in the Li||Cu cells (Supplementary Fig. 3).

Supplementary Note 2

Compatibility with high-voltage cathode

The high-voltage cathode compatibility of HE multi-salt PC electrolyte is also investigated in NCM811 ($LiNi_{0.8}Co_{0.1}Mn_{0.1}O_2$)||Li cells (Supplementary Fig. 4). While a similar initial charge capacity is observed, the $LiPF_6$-PC electrolyte exhibits a slightly lower initial CE of approximately 80%, in contrast to the 83.6% achieved with the HE-PC electrolyte. During the follow cycling test, the capacity of the cells using $LiPF_6$-PC electrolyte fails after 100 cycles whereas the HE-PC electrolyte shows stable cycling with capacity retention of around 93% after 200 cycles. The result indicates that this electrolyte has a better compatibility with the high voltage layered oxide cathodes.

Supplementary Note 3

XPS analysis of SEI on graphite anode

XPS has been conducted to characterize composition of SEI layers formed on the graphite electrode in the $LiPF_6$-PC electrolyte and the HE-PC electrolyte. The SEI derived from the $LiPF_6$-PC electrolyte contains high concentrations of C and O elements, compare to that in the HE-PC electrolyte, indicating a solvent derived SEI. In contrast, a higher content of F, B, N and S elements can be found in SEI from the HE-PC electrolyte, suggesting the decomposition of the anionic group. The detail spectrum in the C 1*s* region contains several peaks (Supplementary Fig. 13). The first peak at 284.8 eV can be attribute to C-C/C-H species. The second peak of C-

O species comes from the decomposition of solvents in the $LiPF_6$-PC electrolyte, while in the HE-PC electrolyte it comes from both solvent and C-O containing salts such as $DFOB^-$. The third peak at around 290.2 eV (assigned to C=O) are from $ROCO_2Li$ formed by the decomposition of PC, where the higher peak intensity of this peaks in the $LiPF_6$-PC electrolyte agrees with more solvent decomposition as compared with the HE-PC electrolyte. Matching the C 1*s* features, signals of O=C (~533 eV) and C-O (~529.5 eV) species are also observed in the O 1*s* spectra (Supplementary Fig. 14), where the $LiPF_6$-PC electrolyte shows higher content of these species than the HE-PC electrolyte. In addition, extra peaks attributed to B-O, N-O, SO_x in the HE-PC electrolyte are observed due to the decomposition of multiple salts (Supplementary Figs. 15-17). In the F 1*s* spectra (Supplementary Fig. 18), peaks due to Li-F (~685.5 eV) and P-F (~687.7 eV) species in both electrolytes are detected due to the decomposition of $LiPF_6$, while in the HE-PC electrolyte the C-F species contributed from other anionic group is also observed together with a decreased content of P-F species. Li-F content in both electrolytes accounted for a large proportion of the F-containing species, which is confirmed in Li-F peak (~56.5 eV) in the Li 1*s* spectra (Supplementary Fig. 19). Hence, by comparing these decomposition species for the $LiPF_6$-PC electrolyte and the HE-PC electrolyte, the relative decomposition relationship between the anion and solvent molecules can be understood.

Supplementary Note 4

Performance with Li-metal anode

Considering the formation of a stable SEI is also an intriguing aspect when examining Li-metal anodes. Hence, the performance of the two electrolytes is assessed through electrochemical evaluation in Li||Cu cells. In comparison to the $LiPF_6$-PC electrolyte, the HE-PC electrolyte demonstrates notably enhanced electrochemical compatibility with Li metal. This is evident in the reversible plating and stripping observed over 200 cycles, showing an average coulombic efficiency (CE) exceeding 99% (Supplementary Fig. 20). Conversely, the cell employing the $LiPF_6$-PC electrolyte falters before reaching 100 cycles. This observation is corroborated by a refined approach[12], where the HE-PC electrolyte achieves an even higher CE of around 99%,

contrasting the approximately 88.5% achieved with the $LiPF_6$-PC electrolyte (Supplementary Fig. 21). Additionally, the overpotential associated with Li plating/stripping is diminished in the HE-PC electrolyte compared to the $LiPF_6$-PC electrolyte, suggesting smoother Li-ion transport facilitated by the inorganic-rich and robust SEI formed in the HE-PC electrolyte.

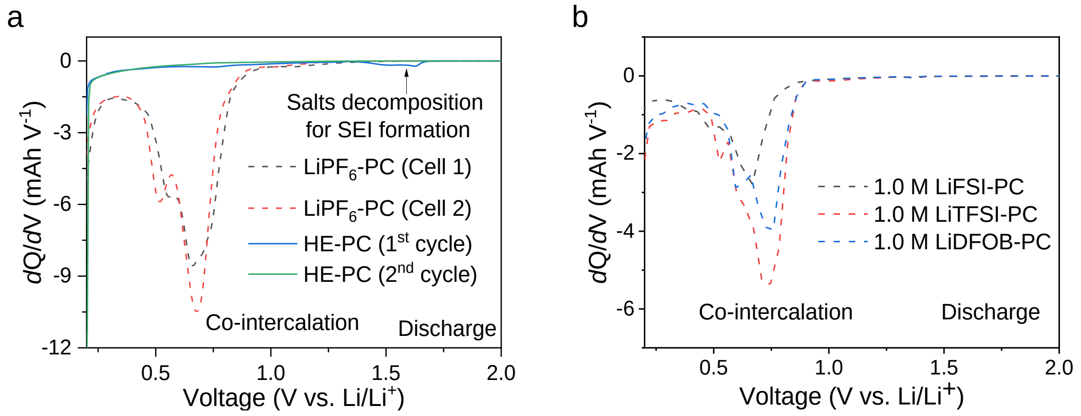

Supplementary Fig. 1. *d*Q/*d*V plots of first lithiation for graphite||Li cells using different electrolytes at 0.1C. a, *d*Q/*d*V plots for graphite||Li cells using single-salt $LiPF_6$-PC and HE-PC electrolytes. Two cells using $LiPF_6$-PC electrolyte shows reduction peak at around 0.7 V vs. Li/Li^+ ascribed to PC co-intercalation. In contrast, the plot for cells using HE-PC electrolyte exhibit smooth curve without co-intercalation peak, and the additional peak at around 1.5 V vs. Li/Li^+ during first discharge, indicating SEI formation that is likely ascribed to salts decomposition. **b**, *d*Q/*d*V plots for the first lithiation of graphite||Li cells using different single salt electrolytes with PC solvent. All these electrolytes show PC co-intercalation peak.

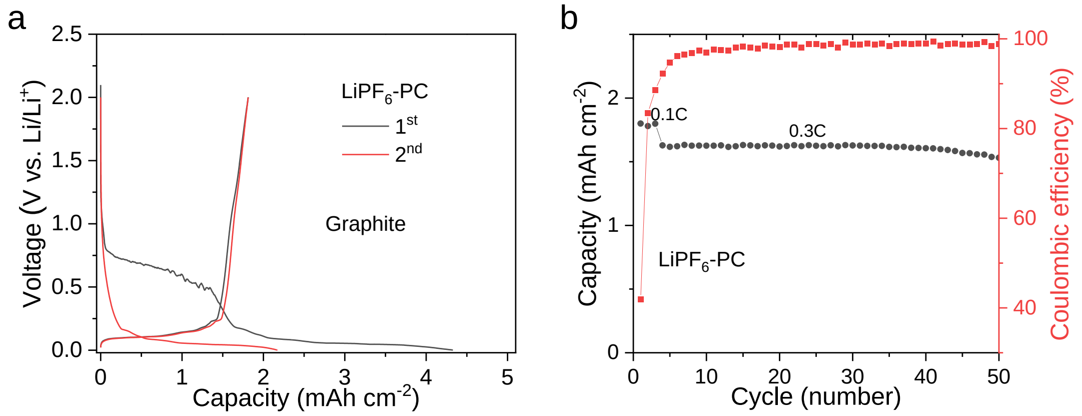

Supplementary Fig. 2. Cycling stability of graphite||Li cell using $LiPF_6$-PC electrolyte. a, The first two cycles voltage profiles of graphite electrodes in the voltage range of 0.001-2.0 V vs. Li/Li^+. The voltage plateau at around 0.7 V corresponds to the PC co-intercalation, leading to high irreversible capacity and consumption of active Li. **b**, Charge capacity retention of graphite||Li cell at 0.1C for the first 3 cycles and 0.3C for the following cycles.

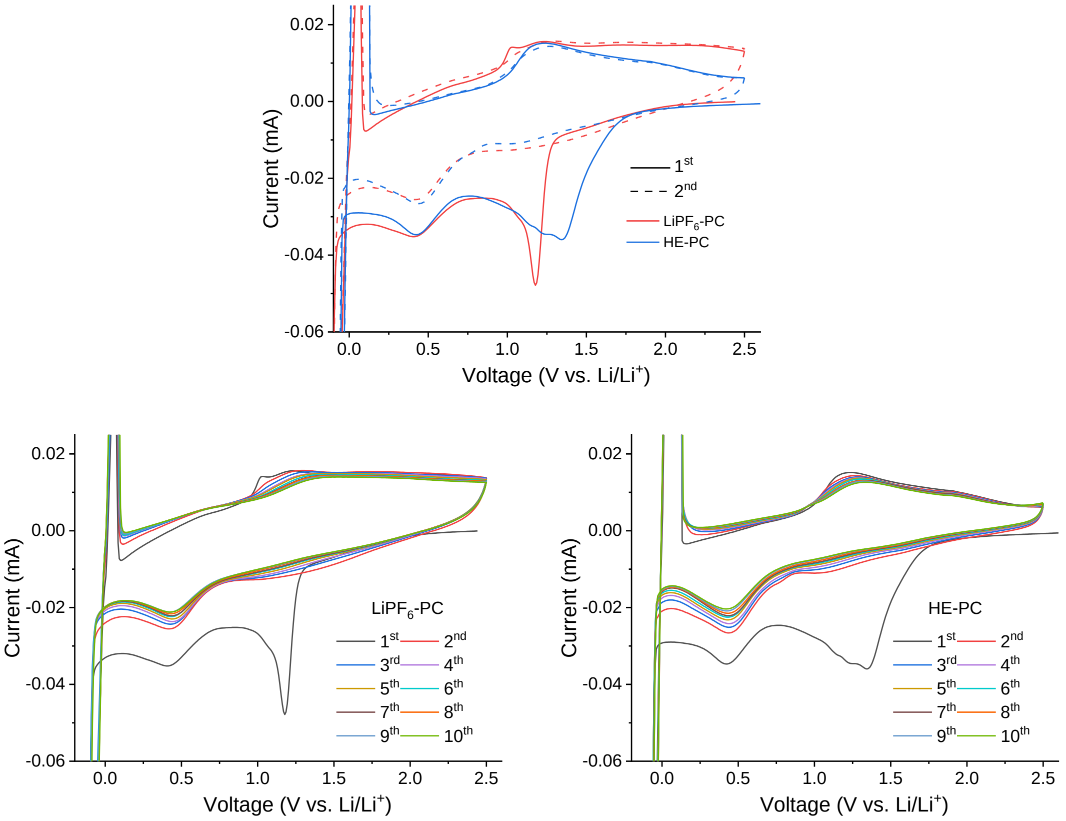

Supplementary Fig. 3. CV curves of Li||Cu cells in a voltage range of -0.1~2.5 V vs. Li/Li$^+$ using single-salt LiPF$_6$-PC and HE-PC electrolytes. To investigate the decomposition of electrolyte at anode side, Li||Cu cells were assembled, taking advantage of the more reactive nature of Li metal compared with graphite.

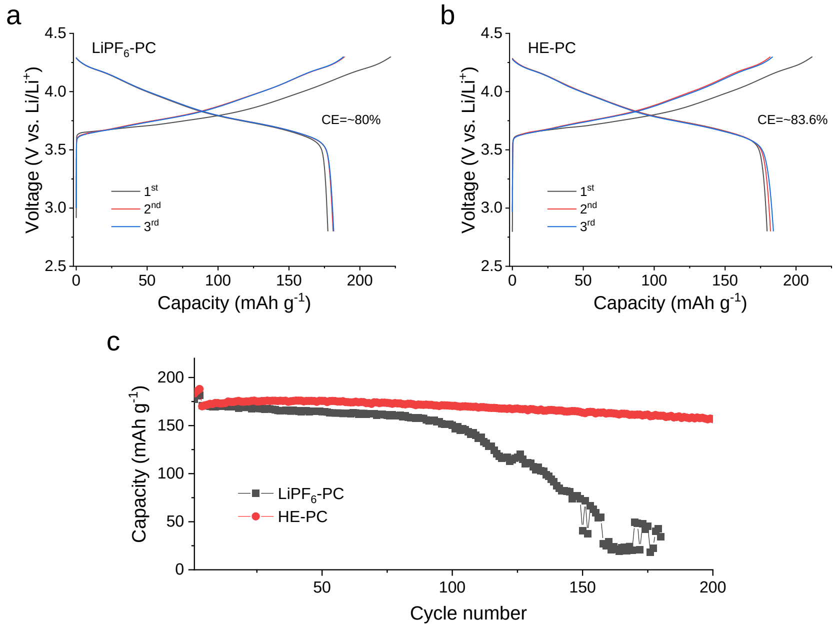

Supplementary Fig. 4. Cycling performance of NCM811||Li cells using different electrolytes. **a**, **b**, Charge/discharge curves of the first three cycles at 0.1C at the voltage range of 2.8-4.3 V. **c**, Cycling stability of NCM811||Li cells at 0.1C for the first three cycles and 1.0C for the following.

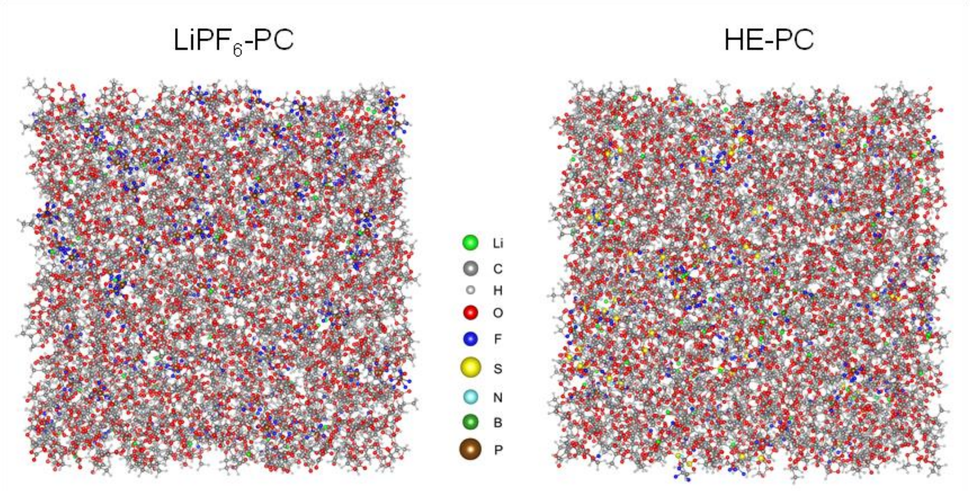

Supplementary Fig. 5. Structure packing of electrolytes for MD simulations. See the method for details.

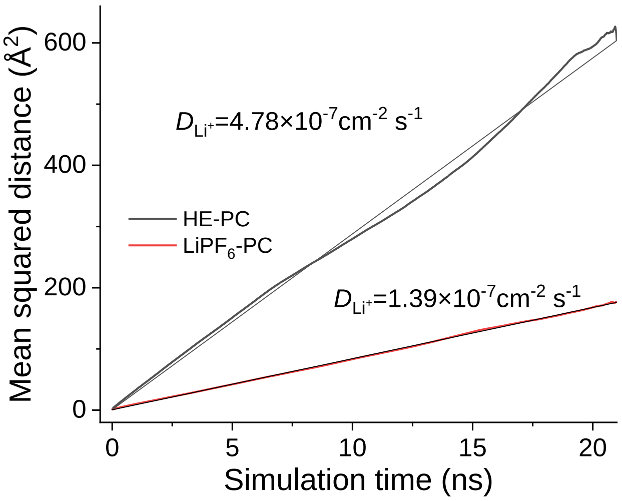

Supplementary Fig. 6. Li^+ self-diffusion coefficient (D_{Li}) from the MD simulated mean squared displacement. The red line is for the $LiPF_6$-PC electrolyte and the black line is for the HE-PC electrolyte.

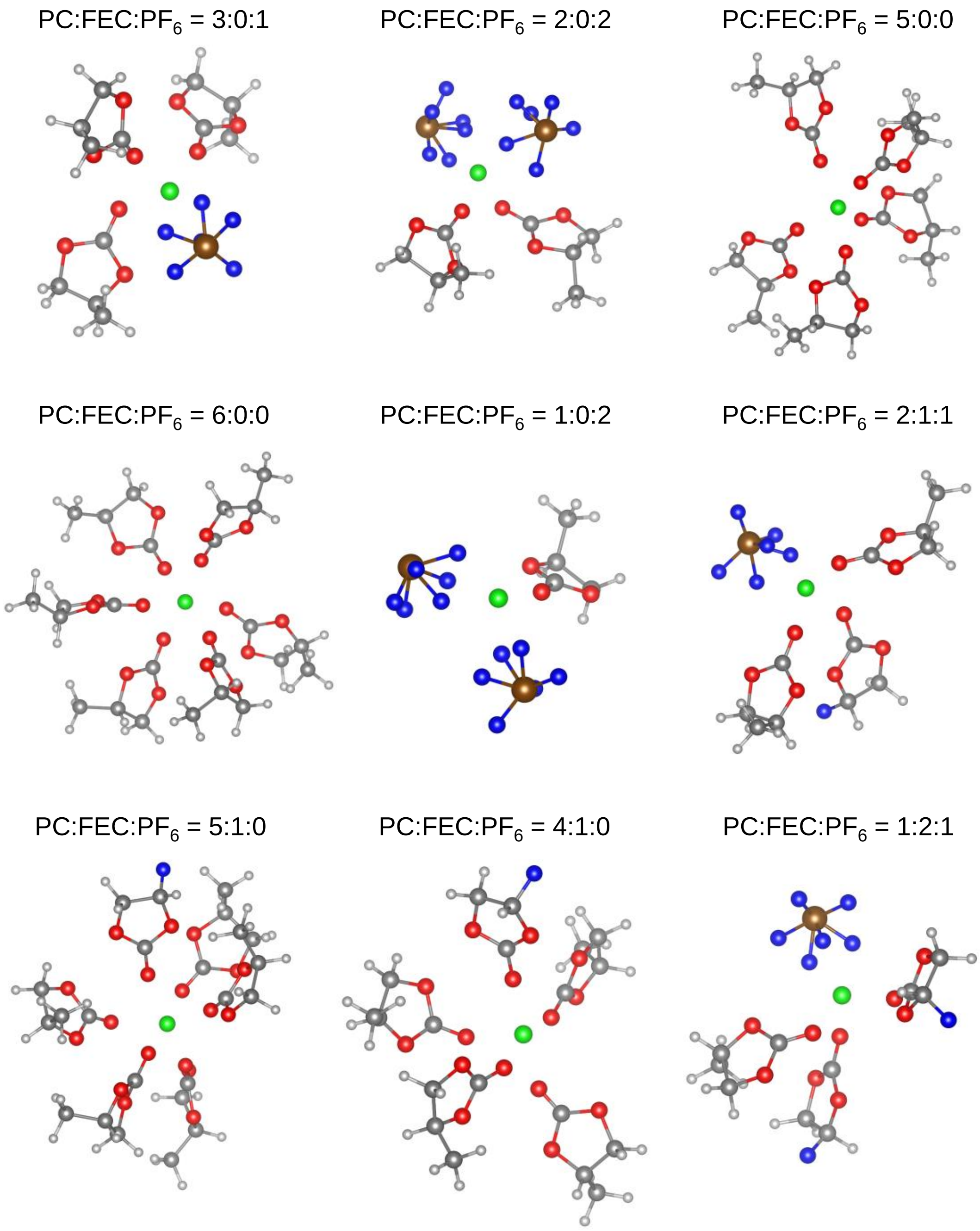

Supplementary Fig. 7. The representative solvation structures in the $LiPF_6$-PC electrolyte.

The detailed structures are shown in Table S1.

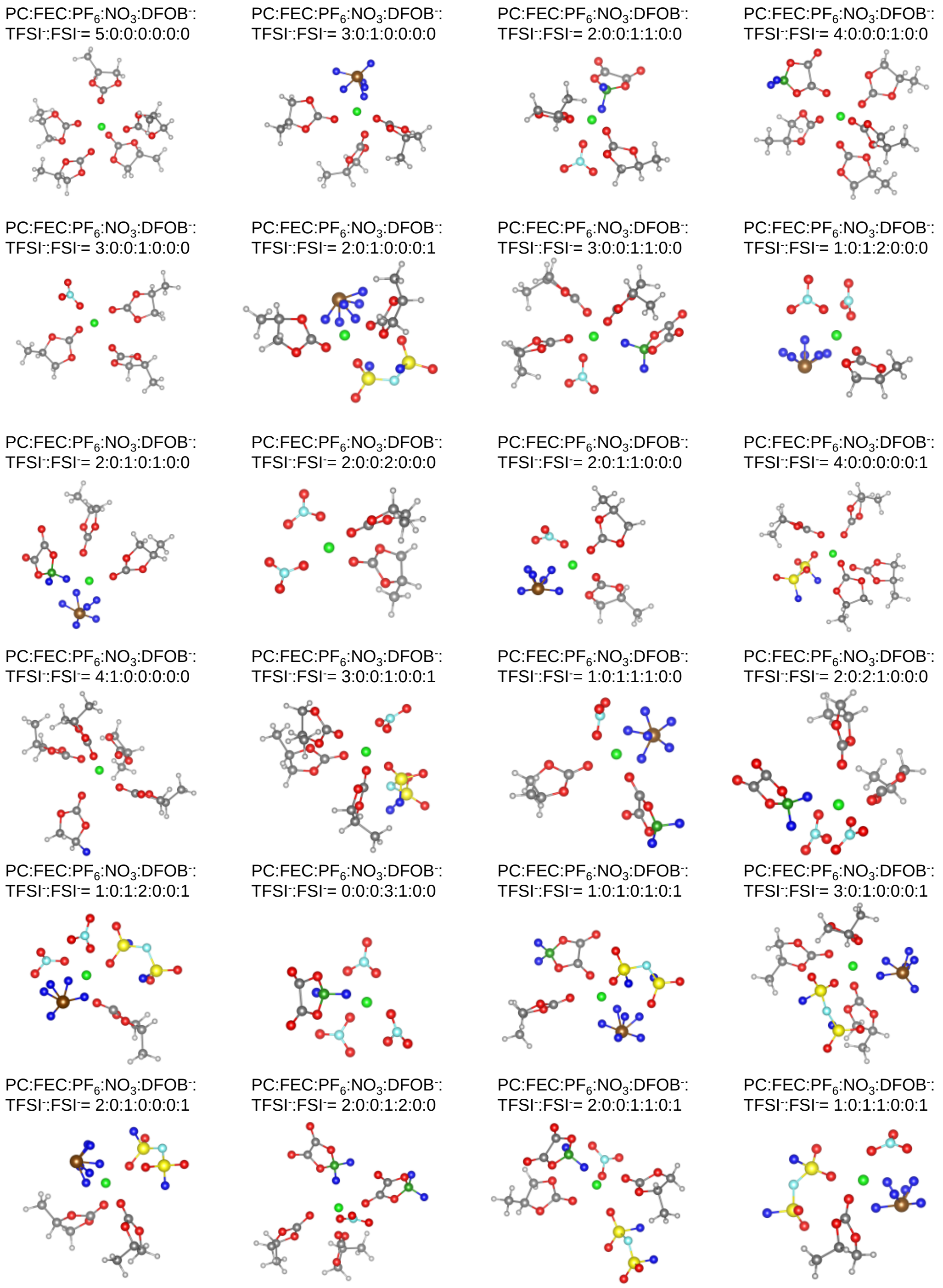

Supplementary Fig. 8. The representative solvation structures in the HE-PC electrolyte.

The detailed structures are shown in Table S2.

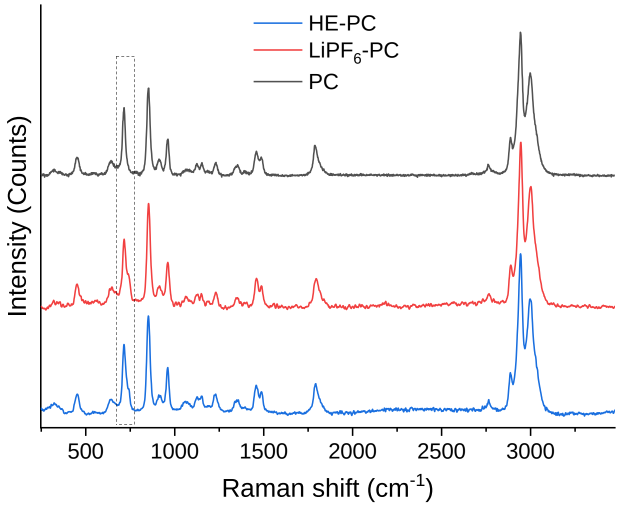

Supplementary Fig. 9. Solvation structure analysis from Raman measurement. Full Raman spectra of PC solvent and different electrolytes.

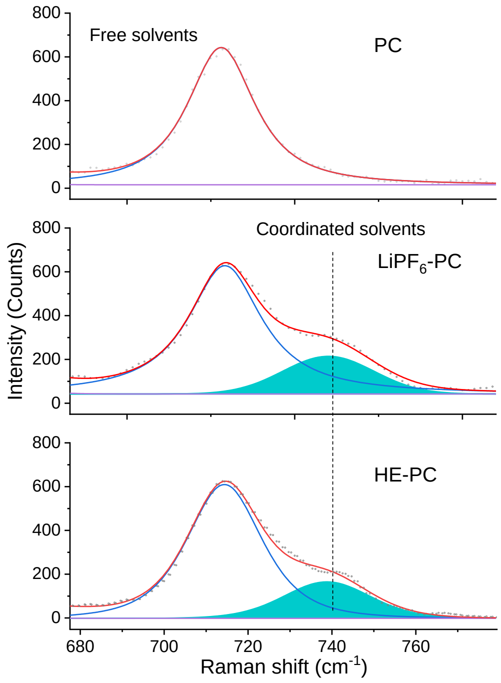

Supplementary Fig. 10. The deconvoluted peaks of the Raman spectra. The $LiPF_6$-PC electrolyte shows larger intensity of the green peak, indicating more coordinated solvent compared to HE-PC electrolyte. The Raman spectra are collected for both electrolytes and the pure PC solvent as shown in Supplementary Fig. 9. The peaks at ~715 cm^{-1} and ~740 cm^{-1} can be attributed to free PC molecules and solvating PC molecules, respectively[13]. With the increase of disorder, the relative contribution of free PC molecules increases and that of solvated PC molecules decreases, indicating decreased interaction between the solvent and the Li^+ in the HE-PC electrolyte.

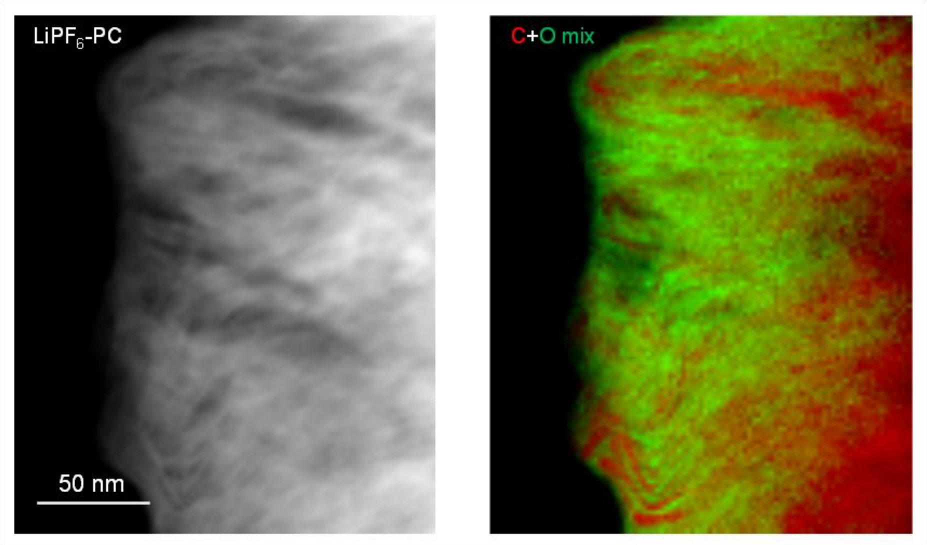

Supplementary Fig. 11. Cryo-STEM EELS of the graphite after PC co-intercalation. The results show that carbon and oxygen can match in the lattice gap, showing solvent distribution.

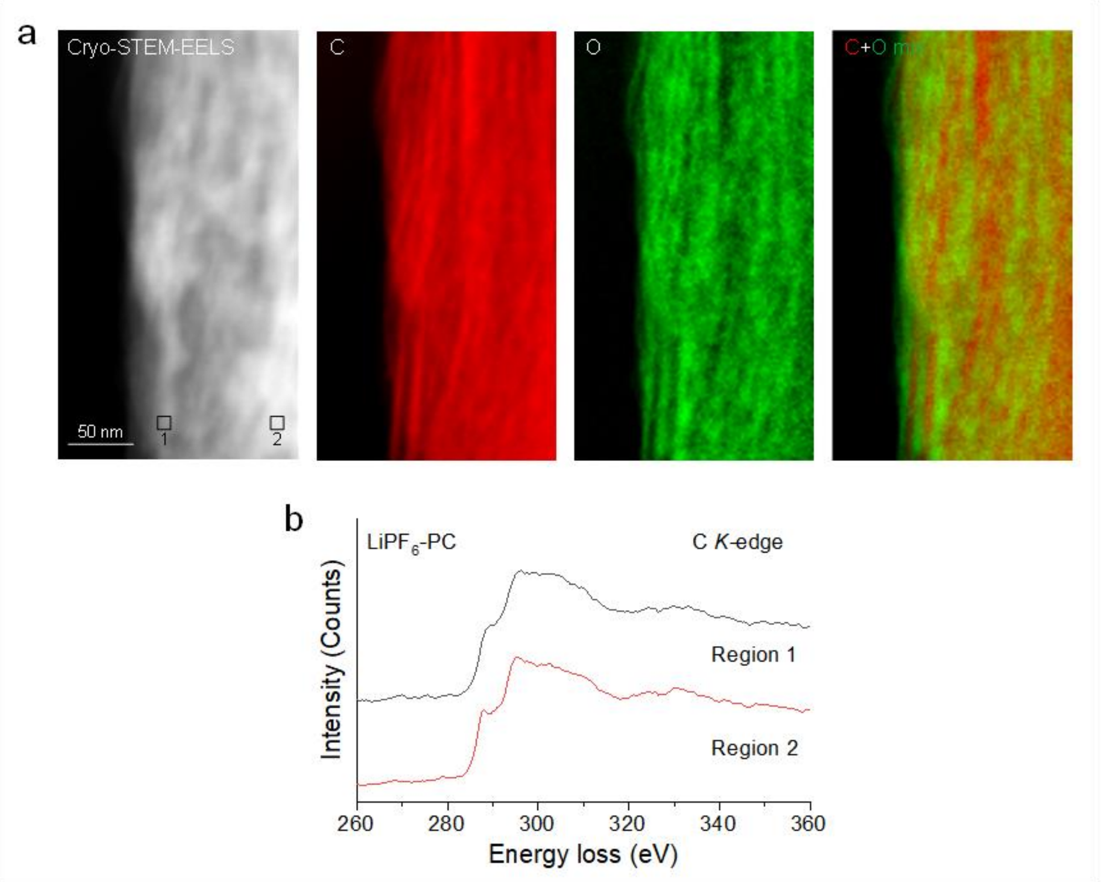

Supplementary Fig. 12. Cryo-STEM EELS of the graphite after PC co-intercalation. a, Cryo-STEM-EELS maps of the graphite in $LiPF_6$-PC electrolyte. **b**, C K-edge fine structure of Region 1 and Region 2 indicated in **a**.

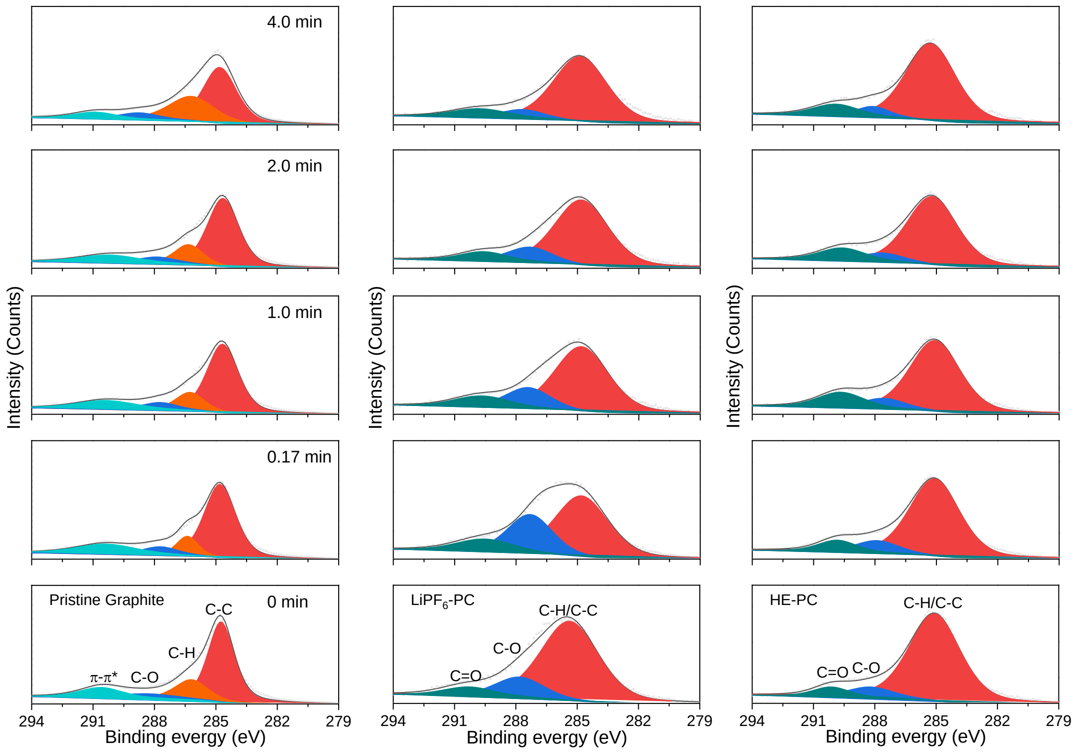

Supplementary Fig. 13. C 1*s* spectra of pristine graphite and SEI on graphite electrodes in electrolytes at different depths.

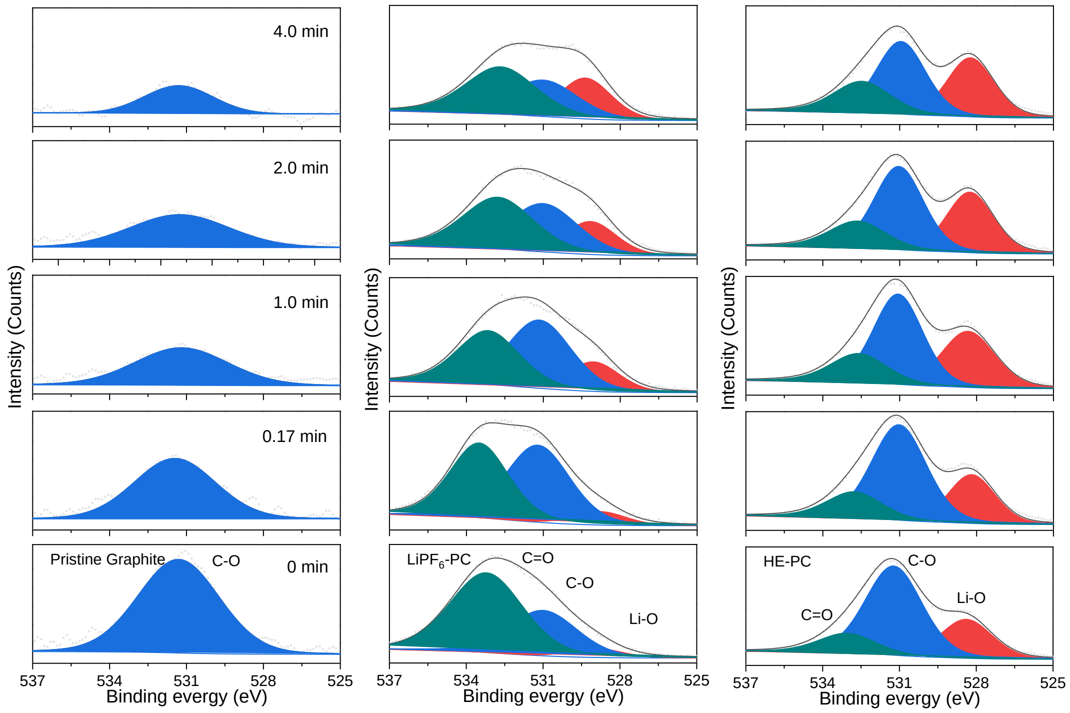

Supplementary Fig. 14. O 1*s* spectra of pristine graphite and SEI on graphite electrodes in electrolytes at different depths.

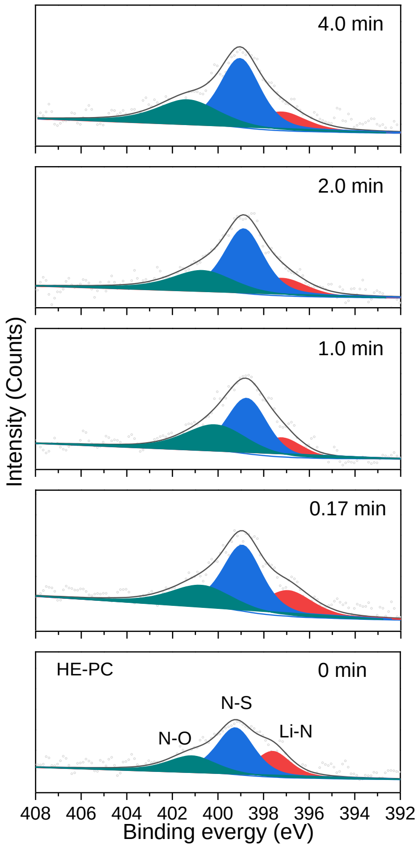

Supplementary Fig. 15. N 1*s* spectra of SEI on graphite electrodes in HE-PC electrolyte at different depths.

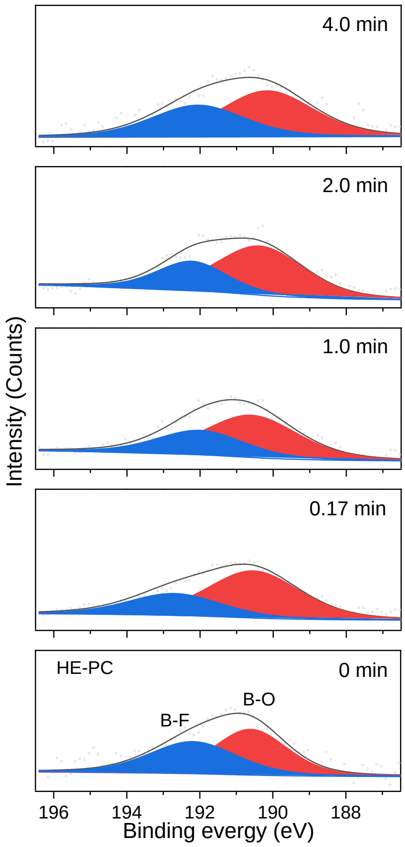

Supplementary Fig. 16. B 1*s* spectra of SEI on graphite electrodes in HE-PC electrolyte at different depths.

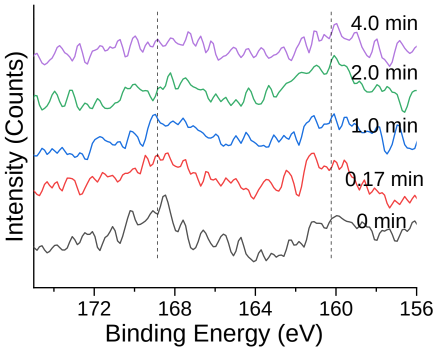

Supplementary Fig. 17. S 2*p* spectra of SEI on graphite electrodes in HE-PC electrolyte at different depths.

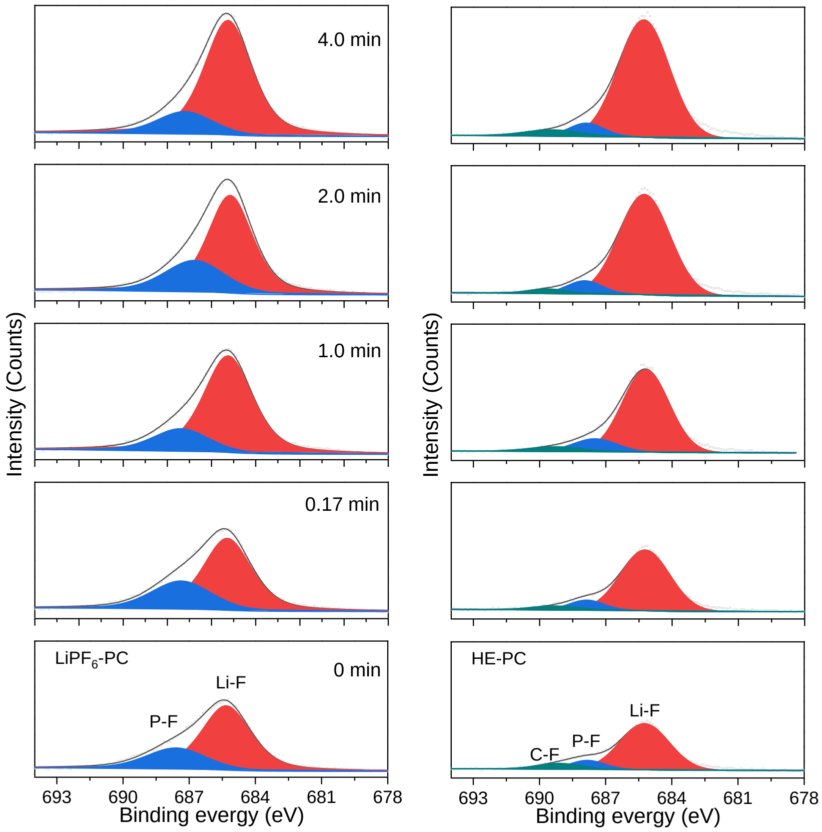

Supplementary Fig. 18. F 1*s* spectra of SEI on graphite electrodes in electrolytes at different depths.

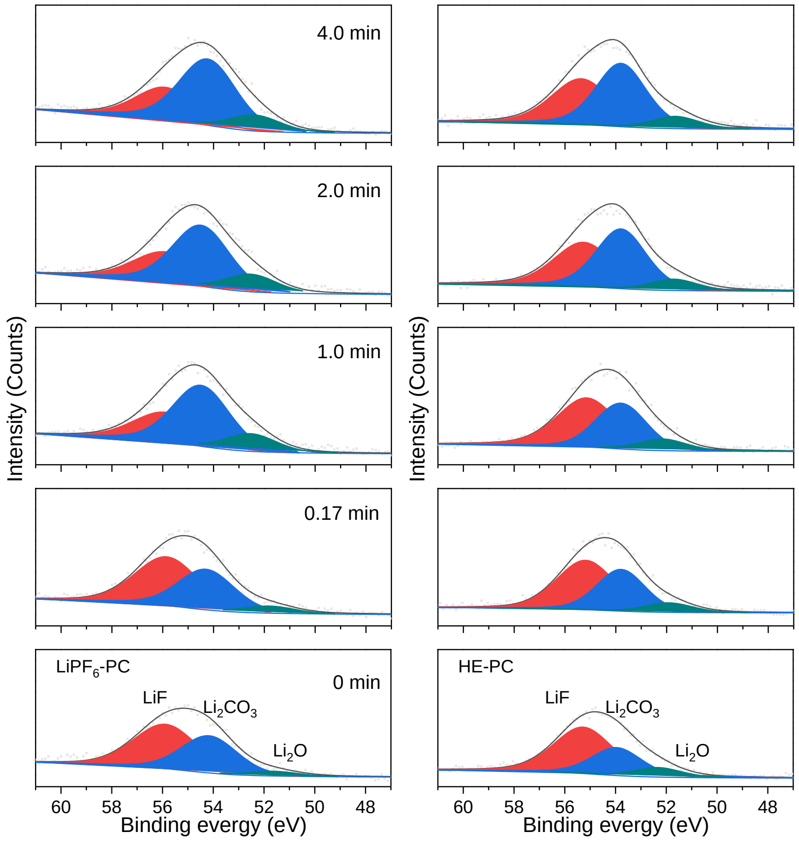

Supplementary Fig. 19. Li 1*s* spectra of SEI on graphite electrodes in electrolytes at different depths.

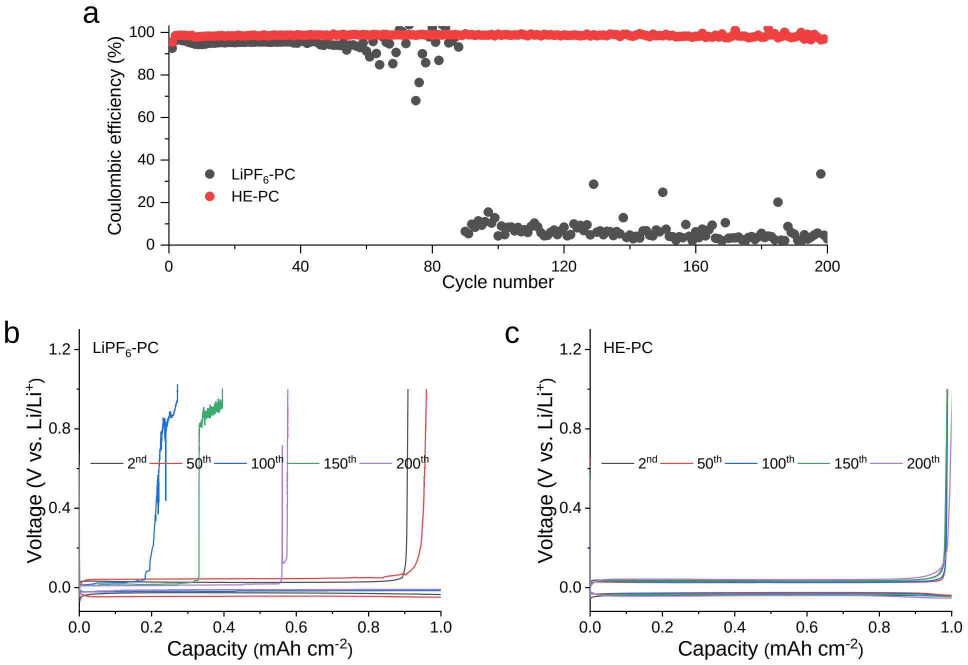

Supplementary Fig. 20. Cycling performance of Li||Cu cells using different electrolytes. a, CEs of Li||Cu cells using different electrolytes. Galvanostatic Li plating/stripping profiles of Li||Cu cells cycled in **b**, $LiPF_6$-PC and **c**, HE-PC electrolytes for selected cycles. Li was electrodeposited at 0.5 mA cm^{-2} to a capacity of 1 mAh cm^{-2}.

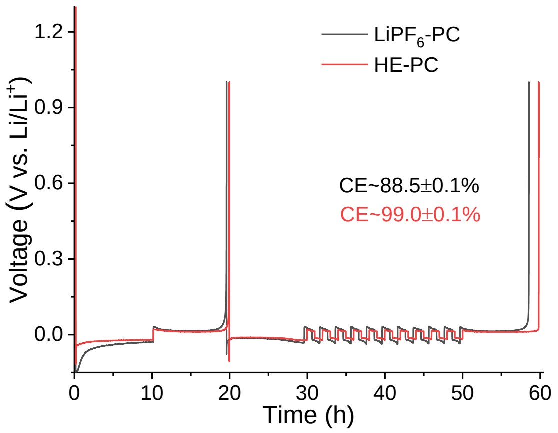

Supplementary Fig. 21. An improved measurement[12, 14] of Li metal CE in Li||Cu cells using different electrolytes. The cell using the HE-PC electrolyte exhibits higher CE around 99.0% and lower overpotential both during initial nucleation and following cycles.

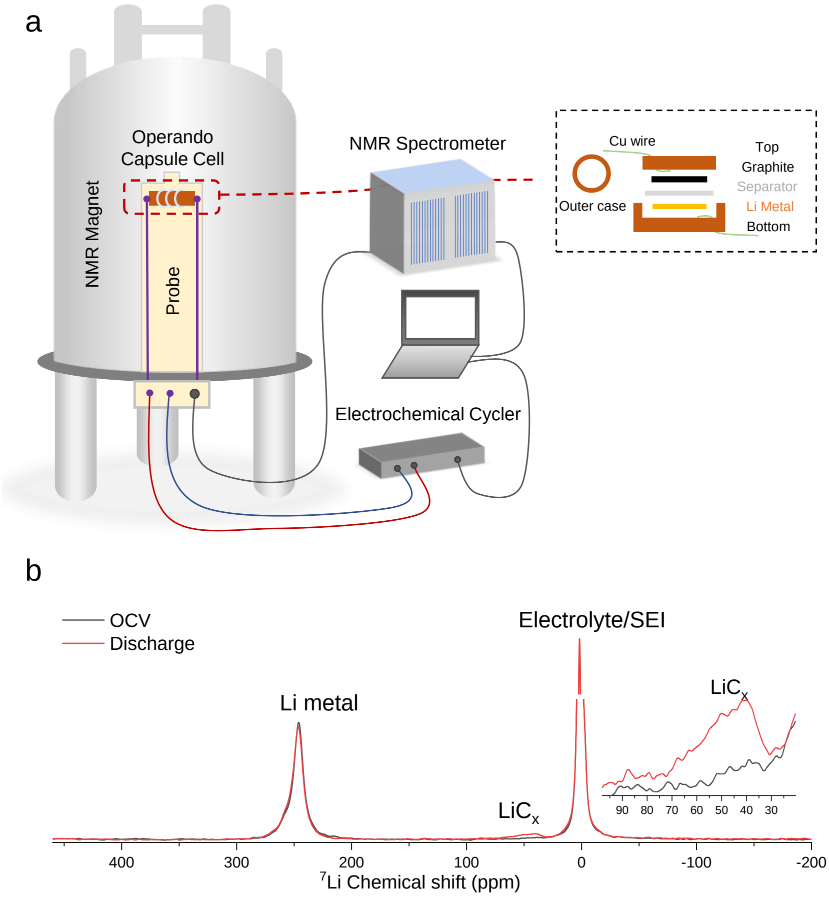

Supplementary Fig. 22. Operando NMR to detect Li intercalation and co-intercalation into graphite. a, The schematic of the operando NMR setup[15]. The dashed box shows the operando capsule cell inserted in the NMR probe coil. The cylindrical casing to assemble electrochemical cells comprising of top, bottom and outer case. Graphite electrode and Li-metal foil counter electrode are connected to copper wires with a separator in between soaked with electrolyte. Then the outer capsule case is used for sealing and producing pressure. The cell is connected to electrochemical cycler for galvanostatic charging/discharging in the NMR magnet. **b**, Representative ^{7}Li NMR spectrum of the operando graphite||Li cell during the formation of LiC_x showing the resonant frequencies of the Li metal, electrolyte and SEI and LiC_x.

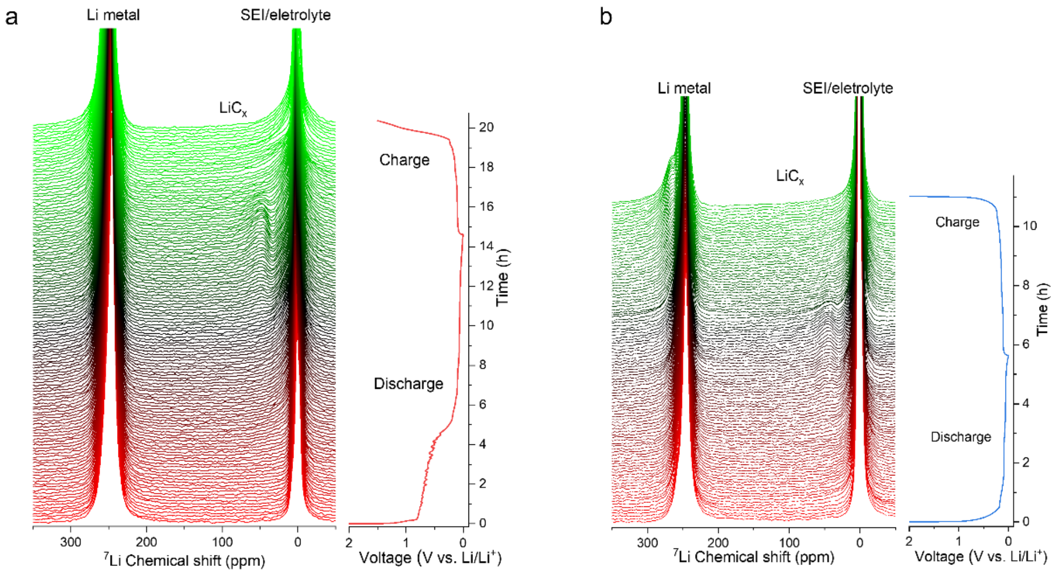

Supplementary Fig. 23. Stacking plot of full ^{7}Li NMR spectra of graphite||Li cells. a is for $LiPF_6$-PC electrolyte and **b** is for HE-PC electrolyte**.**

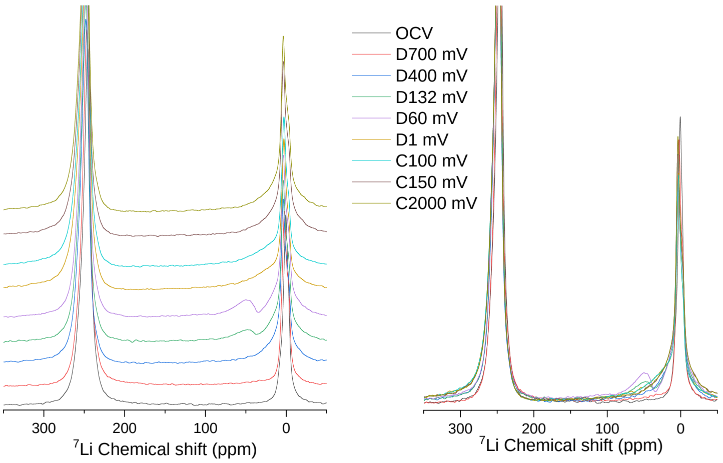

Supplementary Fig. 24. Representative spectra of graphite||Li cell using $LiPF_6$-PC electrolyte extracted from the operando ^{7}Li NMR spectra as indicated by the different voltage.

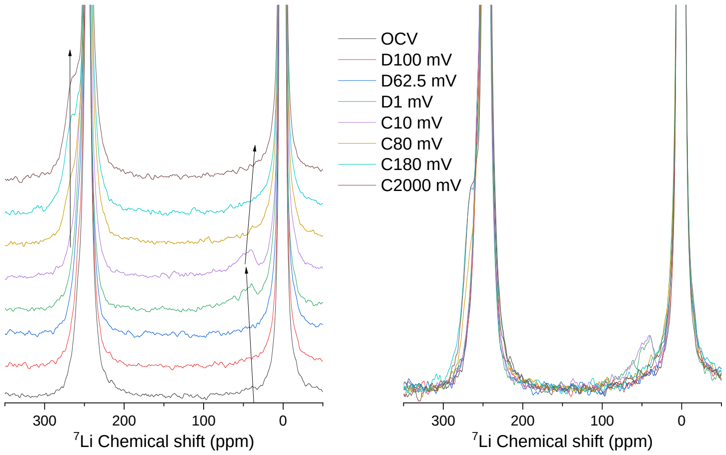

Supplementary Fig. 25. Representative spectra of graphite||Li cell using HE-PC electrolyte extracted from the operando ^{7}Li NMR spectra as indicated by the different voltage.

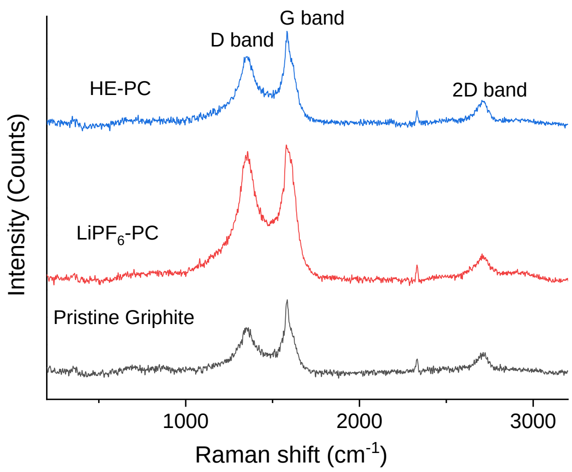

Supplementary Fig. 26. Raman spectra of graphite anode before and after cycling in electrolytes.

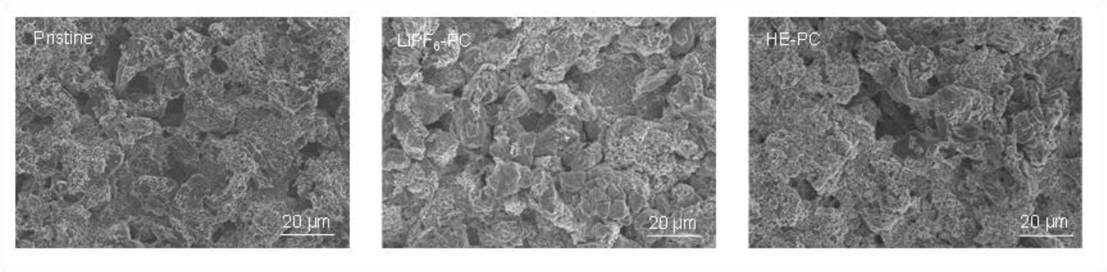

Supplementary Fig. 27. Top-view SEM images at low magnification of graphite anode before and after cycling in different electrolytes.

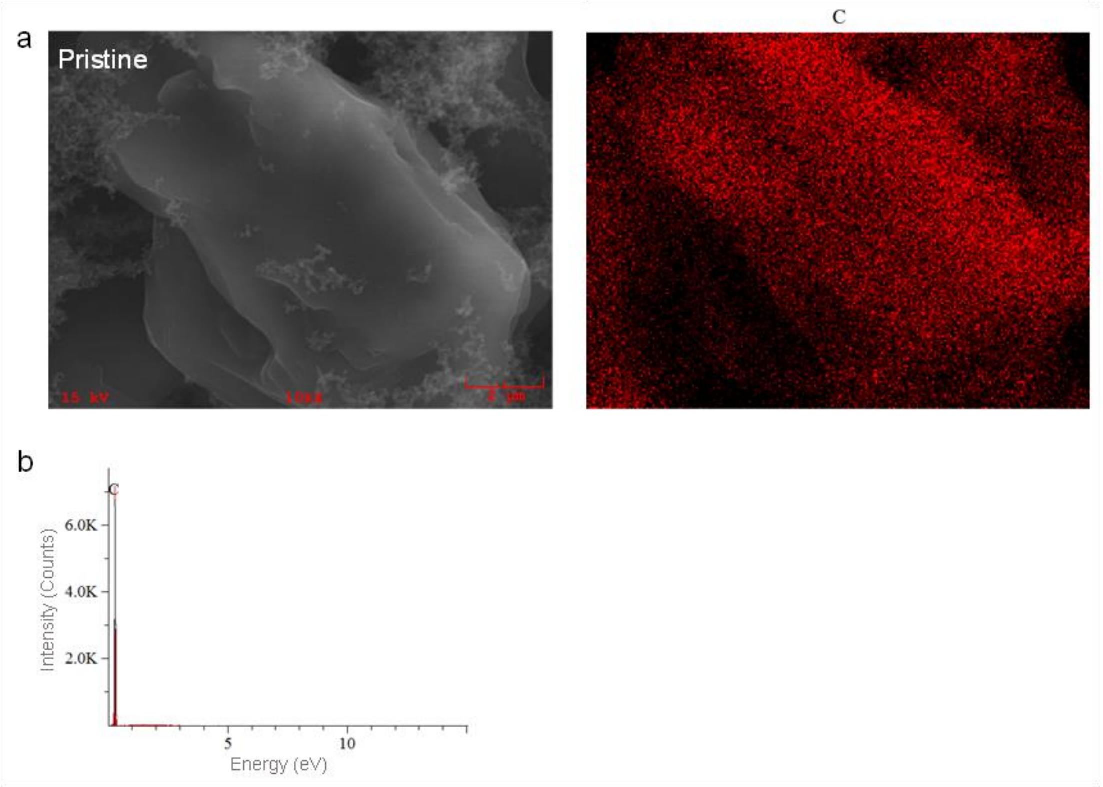

Supplementary Fig. 28. SEM-EDS characterizations of the pristine graphite electrode. a, SEM-EDS mapping for the elemental distributions on pristine graphite electrode. **b**, SEM-EDS spectra in the regions shown in **a**.

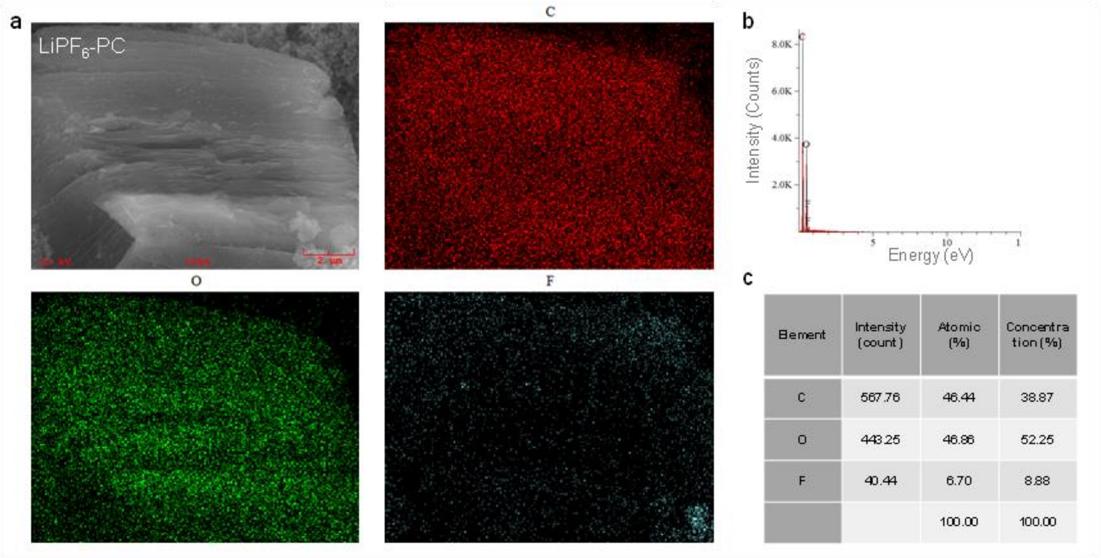

Element	Intensity (count)	Atomic (%)	Concentration (%)
C	567.76	46.44	38.87
O	443.25	46.86	52.25
F	40.44	6.70	8.88
		100.00	100.00

Supplementary Fig. 29. SEM-EDS characterizations of the graphite cycled in $LiPF_6$-PC electrolyte. a, SEM-EDS mapping for the elemental distributions on graphite electrode after one cycle. **b**, SEM-EDS spectra in the regions shown in **a**. **c**, Elemental ratio from SEI based on the SEM-EDS.

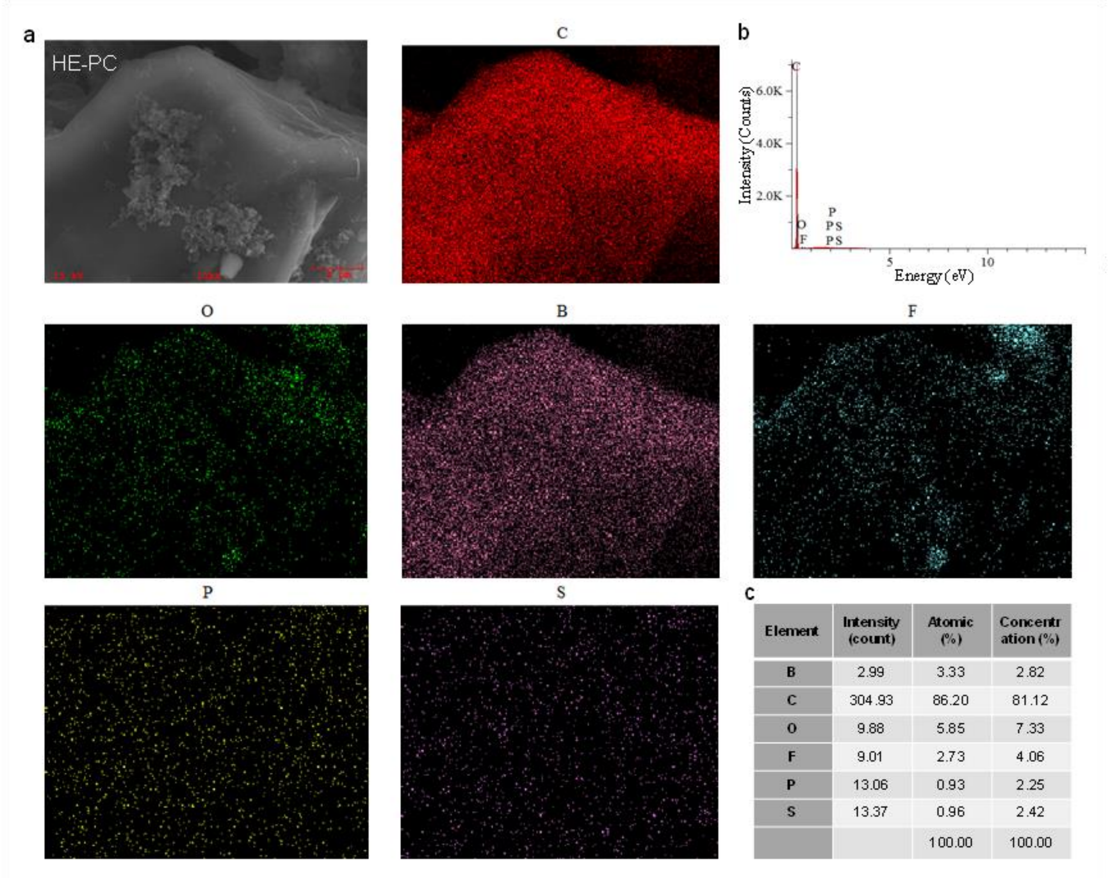

Element	Intensity (count)	Atomic (%)	Concentration (%)
B	2.99	3.33	2.82
C	304.93	86.20	81.12
O	9.88	5.85	7.33
F	9.01	2.73	4.06
P	13.06	0.93	2.25
S	13.37	0.96	2.42
		100.00	100.00

Supplementary Fig. 30. SEM-EDS characterizations of the graphite cycled in HE-PC electrolyte. **a**, SEM-EDS mapping for the elemental distributions on graphite electrode after one cycle. **b**, SEM-EDS spectra in the regions shown in **a**. **c**, Elemental ratio from SEI based on the SEM-EDS.

Supplementary Table 1. The types of solvation structures and their corresponding proportion in the $LiPF_6$-PC electrolyte.

PC	FEC	PF_6^-	Percentage (%)
3	0	1	58.48
2	0	2	15.92
5	0	0	6.77
6	0	0	6.22
1	0	2	4.01
2	1	1	2.88
5	1	0	1.58
4	1	0	1.42
1	2	1	1.11
4	0	0	0.57
2	0	1	0.42
4	0	1	0.38
3	1	1	0.07
3	1	0	0.07
3	0	2	0.04
5	0	1	0.03
4	2	0	0.01
1	1	2	0.01
0	0	2	0.01

Supplementary Table 2. The types of solvation structures and their corresponding proportion in the HE-PC electrolyte.

PC	FEC	PF_6^-	NO_3^-	$DFOB^-$	$TFSI^-$	FSI^-	Percentage (%)
5	0	0	0	0	0	0	9.01
3	0	1	0	0	0	0	8.17
2	0	0	1	1	0	0	5.24
4	0	0	0	1	0	0	4.62
3	0	0	1	0	0	0	4.24
4	0	0	1	0	0	0	4.16
2	0	1	0	0	0	1	3.94
6	0	0	0	0	0	0	3.46
3	0	0	1	1	0	0	3.39
1	0	1	2	0	0	0	3.27
2	0	1	0	1	0	0	3.24
2	0	0	2	0	0	0	3.13
2	0	1	1	0	0	0	3.03
4	0	0	0	0	0	1	2.96
4	1	0	0	0	0	0	1.79
3	0	0	1	0	0	1	1.67
2	0	0	2	1	0	0	1.64
3	0	0	0	0	0	1	1.62
5	1	0	0	0	0	0	1.58
3	1	0	0	0	0	1	1.52
4	0	1	0	0	0	0	1.51
3	0	0	0	0	0	2	1.41
5	0	0	0	1	0	0	1.29
1	0	1	1	1	0	0	1.11
3	0	1	0	0	0	1	1.10
1	0	1	0	1	0	1	1.09

2	0	2	0	0	0	0	1.09
3	0	0	0	1	1	0	1.08
1	0	1	1	0	0	1	1.06
0	0	0	3	1	0	0	1.01
3	0	0	2	0	0	0	0.97
4	0	0	0	0	0	0	0.97
3	0	0	0	1	0	0	0.92
2	0	0	1	0	0	1	0.92
2	0	0	0	0	0	2	0.91
4	0	0	0	0	1	0	0.83
2	1	0	0	1	0	1	0.82
2	0	0	1	2	0	0	0.76
3	0	0	0	2	0	0	0.73
1	0	1	0	0	0	1	0.63
2	0	0	1	1	0	1	0.57
3	0	0	1	0	1	0	0.51
2	0	0	1	0	1	0	0.46
1	0	0	1	1	0	1	0.40
3	1	0	1	0	0	0	0.37
2	0	0	0	2	0	0	0.36
1	0	0	1	2	0	0	0.34
2	1	0	2	0	0	0	0.32
2	2	0	0	0	0	1	0.30
5	0	0	0	0	0	1	0.29
4	0	0	0	0	0	2	0.29
4	2	0	0	0	0	0	0.27
4	0	0	0	1	0	1	0.26
1	0	0	2	1	0	0	0.26
2	1	0	0	0	0	1	0.23

1	1	0	0	1	0	1	0.23
3	0	1	1	0	0	0	0.22
3	0	0	0	1	0	1	0.20
4	1	0	0	0	0	1	0.19
3	2	0	0	0	0	0	0.18
2	0	1	0	0	0	0	0.11
5	0	0	0	0	1	0	0.11
3	1	0	0	0	0	0	0.10
1	0	0	3	1	0	0	0.10
2	0	0	1	1	1	0	0.09
4	0	0	1	1	0	0	0.09
1	0	1	0	1	0	0	0.08
5	0	0	1	0	0	0	0.07
2	0	0	2	0	0	1	0.07
2	0	1	2	0	0	0	0.07
3	0	0	0	0	1	0	0.07
2	0	0	0	1	1	0	0.06
3	0	0	2	1	0	0	0.06
1	1	0	2	0	0	0	0.06
3	1	0	0	1	0	1	0.04
4	0	0	1	0	0	1	0.04
2	1	0	2	1	0	0	0.04
2	1	0	1	1	0	0	0.04
3	1	0	0	0	1	0	0.04
4	1	0	0	0	1	0	0.04
4	0	0	0	1	1	0	0.03
4	0	0	2	0	0	0	0.03
1	0	2	0	0	0	0	0.03
2	0	0	0	0	0	1	0.03

2	0	0	1	0	0	0	0.03
3	0	0	2	0	0	1	0.02
3	1	1	0	0	0	0	0.02
4	1	1	0	0	0	0	0.02
1	1	0	0	0	0	1	0.02
1	0	1	1	0	0	0	0.02
5	0	1	0	0	0	0	0.02
1	0	1	2	0	0	1	0.02
1	1	0	0	2	0	0	0.01
1	2	0	0	0	0	1	0.01
2	1	0	0	2	0	0	0.01
2	1	0	1	0	0	0	0.01
2	0	1	1	0	0	1	0.01
3	1	0	0	1	0	0	0.01
3	2	0	0	0	0	1	0.01
2	0	0	0	1	0	0	0.01
0	0	1	1	0	0	1	0.01
4	0	0	1	0	1	0	0.01
2	2	0	0	0	0	0	0.01
3	0	1	0	1	0	0	0.01
7	0	0	0	0	0	0	0.01
1	0	0	1	1	0	0	0.01
1	0	0	0	0	0	2	0.01

References

1. Kim, S. C.; Kong, X.; Vilá, R. A.; Huang, W.; Chen, Y.; Boyle, D. T.; Yu, Z.; Wang, H.; Bao, Z.; Qin, J.; Cui, Y., Potentiometric Measurement to Probe Solvation Energy and Its Correlation to Lithium Battery Cyclability. *Journal of the American Chemical Society* **2021,** *143* (27), 10301-10308.
2. Van Der Spoel, D.; Lindahl, E.; Hess, B.; Groenhof, G.; Mark, A. E.; Berendsen, H. J. C., GROMACS: Fast, flexible, and free. *Journal of Computational Chemistry* **2005,** *26* (16), 1701-1718.
3. Berendsen, H. J. C.; van der Spoel, D.; van Drunen, R., GROMACS: A message-passing parallel molecular dynamics implementation. *Computer Physics Communications* **1995,** *91* (1), 43-56.
4. Lindahl, E.; Hess, B.; van der Spoel, D., GROMACS 3.0: a package for molecular simulation and trajectory analysis. *Molecular modeling annual* **2001,** *7* (8), 306-317.
5. Abraham, M. J.; Murtola, T.; Schulz, R.; Páll, S.; Smith, J. C.; Hess, B.; Lindahl, E., GROMACS: High performance molecular simulations through multi-level parallelism from laptops to supercomputers. *SoftwareX* **2015,** *1-2*, 19-25.
6. Wang, J.; Wolf, R. M.; Caldwell, J. W.; Kollman, P. A.; Case, D. A., Development and testing of a general amber force field. *Journal of Computational Chemistry* **2004,** *25* (9), 1157-1174.
7. Sousa da Silva, A. W.; Vranken, W. F., ACPYPE - AnteChamber PYthon Parser interfacE. *BMC Research Notes* **2012,** *5* (1), 367.
8. Sambasivarao, S. V.; Acevedo, O., Development of OPLS-AA Force Field Parameters for 68 Unique Ionic Liquids. *Journal of Chemical Theory and Computation* **2009,** *5* (4), 1038-1050.
9. Martínez, L.; Andrade, R.; Birgin, E. G.; Martínez, J. M., PACKMOL: A package for building initial configurations for molecular dynamics simulations. *Journal of Computational Chemistry* **2009,** *30* (13), 2157-2164.
10. Gowers, R.; Linke, M.; Barnoud, J.; Reddy, T.; Melo, M.; Seyler, S. L.; Dotson, D.; Domanski, J.; Buchoux, S.; Kenney, I., MDAnalysis: a Python package for the rapid analysis of molecular dynamics simulations. In *Proceedings of the 15th Python in Science Conference*, Austin, TX, 2016.
11. Zhao, H.; Park, S.-J.; Shi, F.; Fu, Y.; Battaglia, V.; Ross, P. N.; Liu, G., Propylene Carbonate (PC)-Based Electrolytes with High Coulombic Efficiency for Lithium-Ion Batteries. *Journal of The Electrochemical Society* **2013,** *161* (1), A194-A200.
12. Aurbach, D.; Gofer, Y.; Langzam, J., The Correlation Between Surface Chemistry, Surface Morphology, and Cycling Efficiency of Lithium Electrodes in a Few Polar Aprotic Systems. *Journal of The Electrochemical Society* **1989,** *136* (11), 3198-3205.
13. Jeong, S.-K.; Inaba, M.; Iriyama, Y.; Abe, T.; Ogumi, Z., Electrochemical Intercalation of Lithium Ion within Graphite from Propylene Carbonate Solutions. *Electrochemical and Solid-State Letters* **2003,** *6* (1), A13.
14. Adams, B. D.; Zheng, J.; Ren, X.; Xu, W.; Zhang, J.-G., Accurate Determination of Coulombic Efficiency for Lithium Metal Anodes and Lithium Metal Batteries. *Advanced Energy Materials* **2018,** *8* (7), 1702097.
15. Wang, Q.; Zhao, C.; Wang, S.; Wang, J.; Liu, M.; Ganapathy, S.; Bai, X.; Li, B.; Wagemaker, M., Clarifying the Relationship between the Lithium Deposition Coverage and Microstructure in Lithium Metal Batteries. *Journal of the American Chemical Society* **2022,** *144* (48), 21961-21971.
